# Age-related shifts of dissolved hydrogen and its impact on rumen fermentation dynamics and microbial communities in goats (*Capra hircus*)

**DOI:** 10.1186/s12866-026-05563-x

**Published:** 2026-08-27

**Authors:** Weiwei Wang, You Tian, Wei Guo, Yuntao Dong, Jiandui Mi, Anum Ali Ahmad, Xiaoping Jing, Ruijun Long, Xuezhao Sun, Qing Deng, Lizhuang Hao, Xiaoyang Lv, Wei Sun, Xiang Chen

**Affiliations:** 1 Laboratory of Animal Genetics, Breeding and Reproduction in the Plateau Mountainous Region, Ministry of Education, College of Animal Science, Guizhou University, Guiyang, 550025 China; 2 State Key Laboratory for Animal Disease Control and Prevention, College of Veterinary Medicine, Lanzhou University, Lanzhou, 730000 China; 3 The Roslin Institute and Royal (Dick) School of Veterinary Studies, University of Edinburgh, Edinburgh, EH25 9RG UK; 4 Research Center for the Belt and Road, Lanzhou University, Lanzhou, 730000 China; 5 Bioeconomy Science Institute, Grasslands Research Centre, Palmerston North, New Zealand; 6 Key Laboratory of Plateau Grazing Animal Nutrition and Feed Science of Qinghai Province, Qinghai University, Xining, China; 7 Joint International Research Laboratory of Agriculture and Agri-Product Safety of Ministry of Education, Yangzhou University, Yangzhou, 225002 China; 8 International Joint Research Laboratory in Universities of Jiangsu Province for Domestic Animal Germplasm Resources and Genetic Improvement, Yangzhou University, Yangzhou, 225002 China

**Keywords:** Dissolved hydrogen shifting, Rumen microbes, Dissolved methane, Gibbs free energy, Microbial metabolic pathways

## Abstract

The rumen is a complex anaerobic fermentation ecosystem in which microbial communities undergo dynamic changes during host development, influencing fermentation efficiency, hydrogen utilization, and methane (CH₄) production. However, how rumen maturation reshapes hydrogen redistribution and the associated microbial interactions remains poorly understood. This study compared rumen fermentation characteristics, microbial communities (bacteria, archaea, fungi, and protozoa), and Gibbs free energy profiles between 0.5-year-old and 2-year-old goats to elucidate age-related changes in hydrogen metabolism. Adult goats exhibited significantly higher concentrations of dissolved methane (dCH₄), dissolved hydrogen (dH₂), and total short-chain fatty acids (SCFAs), including acetate, propionate, and butyrate, than young goats (*P* < 0.05). Thermodynamic analyses indicated that glucose fermentation and methanogenesis were energetically feasible under rumen conditions (ΔG < 0), while age-related differences in Gibbs free energy (ΔG) suggested altered hydrogen partitioning during rumen maturation. The mature rumen was characterized by enrichment of carbohydrate-fermenting microorganisms, particularly *Segatella*, *Anaeroplasma*, and the anaerobic fungus *Cyllamyces*, which were positively associated with glucose conversion pathways, metabolic hydrogen, and SCFA production. In contrast, younger goats showed enrichment of amino acid metabolism pathways together with microbial taxa associated with early rumen development, including *Christensenellaceae_R-7_group* and *Caecomyces*. The archaeal community also shifted with age, with significantly higher abundances of *Methanosphaera* and *Methanosarcina* in adult goats, consistent with enhanced hydrogen utilization through methanogenic pathways. Functional predictions further revealed enrichment of carbohydrate and energy metabolism in adult goats, whereas amino acid metabolism predominated in young goats. These findings demonstrate that rumen maturation is accompanied by coordinated changes in microbial community structure, metabolic hydrogen redistribution, and thermodynamic characteristics, resulting in a transition from amino acid-oriented metabolism in young goats to a more specialized carbohydrate-fermenting ecosystem in adults. This integrated analysis identifies key microbial groups involved in hydrogen turnover and may provide new insights into the microbial mechanisms underlying rumen maturation, offering potential targets for improving feed efficiency and mitigating methane emissions in ruminants.

**Supplementary Information:**

The online version contains supplementary material available at https://doi.org/10.1186/s12866-026-05563-x.

## Introduction

Ruminants play a crucial role in global food systems because of their unique ability to convert low-quality fibrous feed into high-value animal products such as meat and milk. This capacity relies on the complex rumen microbial ecosystem, which supplies up to 70% of the host’s energy and protein requirements through the fermentation of plant carbohydrates into short-chain fatty acids (SCFAs) [1]. During fermentation, metabolic hydrogen ([2 H]) serves as a central link between microbial metabolic processes, fermentation pathways, and methane (CH₄) production. In the rumen, it is generated as reducing equivalents and can be transferred to molecular H₂, which exists in both dissolved and gaseous forms. Dissolved H₂ is the form directly available for microbial utilisation and metabolism, representing 100% of the bioavailable H₂ pool [2–4]. Metabolic hydrogen is partitioned among competing electron sinks, with methanogenesis representing the predominant route of hydrogen disposal. Alternative pathways, including propionate formation and reductive acetogenesis, can improve fermentation efficiency and reduce methane formation [5, 6]. Because enteric methane has a global warming potential approximately 28 times that of carbon dioxide and represents an energy loss of 2–15% of gross energy intake in ruminants [7, 8], redirecting reducing equivalents away from methanogenesis could be an important strategy for improving feed efficiency and mitigating greenhouse gas emissions.

Although diet is the primary factor shaping rumen microbial composition and fermentation [9], host age also plays a fundamental role in rumen development. During early life, the rumen undergoes rapid microbial colonisation and ecological succession, resulting in marked changes in microbial composition, substrate utilisation, and fermentation characteristics [10–13]. As the rumen matures, microbial communities become increasingly stable and functionally specialised, with greater capacity for fibre degradation and energy harvesting [12, 13]. These developmental changes are expected to influence the production, transfer, and utilisation of hydrogen, thereby altering electron flow, fermentation efficiency, and methanogenic activity.

Despite extensive studies describing age-related changes in rumen microbial communities and fermentation characteristics [10–12], the mechanisms by which rumen maturation regulates hydrogen redistribution remain poorly understood. Most previous studies have focused on microbial succession, fermentation products, or methane production individually [14, 15], whereas the interactions between hydrogen metabolism, microbial community dynamics, and thermodynamic characteristics have received comparatively little attention. In particular, the coordinated roles of bacteria, archaea, anaerobic fungi, and protozoa in regulating hydrogen production, interspecies hydrogen transfer, and hydrogen utilization during rumen maturation have not been comprehensively investigated [6, 16]. Understanding these microbial interactions is essential for elucidating how rumen development influences fermentation efficiency and methanogenic potential.

We therefore hypothesized that rumen maturation reshapes dissolved hydrogen redistribution through coordinated changes in the rumen microbiome, leading to differences in fermentation characteristics and methanogenic potential between young and adult goats. To test this hypothesis, we compared rumen fermentation characteristics, microbial communities (bacteria, archaea, fungi, and protozoa), metabolic hydrogen redistribution, and Gibbs free energy profiles between 0.5-year-old and 2-year-old goats. Unlike previous studies that examined microbial composition or fermentation characteristics separately, this study combines microbial ecology, hydrogen partitioning and thermodynamic analyses to provide an integrated understanding of age-related changes in rumen hydrogen metabolism. These findings may offer new insights into the microbial mechanisms underlying rumen maturation and provide a scientific basis for developing microbiome-based strategies to improve rumen fermentation efficiency and mitigate methane emissions.

## Materials and methods

### Study site, animals, and feed

The study was conducted at the Fuxing Muye Research Station (1,125 m altitude; 28°15’26.2"N and 106°11’58.1"E), a research station of Guizhou University located in Xishui County, Guizhou Province, China. All experimental goats were provided by the research station. Twelve healthy Qianbei Ma male goats (wethers) were divided into two age groups (*n* = 6 per group): a 0.5-year-old group (average body weight, 25.4 ± 1.9 kg) and a 2-year-old group (average body weight, 42.7 ± 2.1 kg). They were housed in individual pens. All animals were fed the same total mixed ration (TMR) consisting of corn silage, Chinese wild rye (*Secale sylvestre*), corn, soybean meal, wheat bran, calcium carbonate, sodium chloride, and a premix. Feed was offered twice daily at 9:00 and 17:00, with free access to water. The chemical composition of the diet was as follows: dry matter (648 g/kg), crude protein (162 g/kg), neutral detergent fiber (349 g/kg), acid detergent fiber (173 g/kg), and metabolizable energy (10.3 MJ/kg DM). All components except metabolizable energy were determined by direct measurement. Metabolizable energy (ME) was calculated according to the Chinese Agricultural Industry Standard Nutrient Requirements of Meat Sheep (NY/T 816–2021) [17]. Animals were acclimated to housing and diet for one month before sampling. During this period, average daily dry matter intake (DMI) and average daily gain (ADG) were recorded (Supplementary Table 1).

### Sampling procedures and analytical methods

#### Rumen fluid collection and gas sampling

The animals were fed at 17:00, refusals were removed at 18:00 while water remained available. Rumen fluid samples were collected before the morning feeding the next day. Each goat was gently restrained, and a clean rigid plastic mouth pipe (2.5 cm in diameter) was inserted, followed by a stomach tube (120 cm in length, 1.5 cm external diameter) attached to a vacuum sampler. The initial 20–30 mL of rumen fluid was discarded to avoid saliva contamination before collecting 100 mL from each animal.

Briefly, each rumen fluid sample (35 mL) was rapidly transferred into a 50-mL plastic syringe (Kangdelai, Shanghai, China). The 50-mL syringe containing the rumen fluid was connected via a T-valve to a 20-mL syringe pre-filled with 10 mL of N₂ gas. The N₂ gas was injected from the 20-mL syringe into the 50-mL syringe through the T-valve, and the connected syringes were then vigorously shaken by hand for 5 min to facilitate the extraction of dissolved gases from the rumen fluid. The extracted gas (less than 10 mL) was subsequently transferred into a 10-mL vacuum tube (Xinle, China) for analysis. Changes in the volume readings of both syringes, together with the room temperature, were recorded as previously described [18]. Rumen pH was measured using a calibrated portable pH meter (Sartorius PB-10, Goettingen, Germany). Aliquots for SCFA analysis were stored at − 80 °C until further analysis.

#### Chemical analyses

Feed samples were analyzed for dry matter (DM; AOAC 930.15), crude protein (CP; AOAC 984.13), ether extract (EE; AOAC 920.39), and ash (AOAC 942.05) according to AOAC [19]. Neutral detergent fiber (NDF) and acid detergent fiber (ADF) were determined following Van Soest et al. [20].

Short-chain fatty acids (SCFAs) were quantified using a gas chromatograph (Agilent Technologies, 7890B) equipped with a flame ionization detector and an AT-FFAP type capillary column (30 m × 0.32 mm × 0.5 μm). Analytical conditions followed Erwin et al. [21], Wang et al. [22] and Zhang et al. [23].

Gaseous CH₄ and H₂ were determined following Wang et al. [22]. A 100 µL gas sample was injected into a gas chromatograph equipped with a 13 × molecular sieve packed column and a thermal conductivity detector (TCD). Column and detector temperatures were maintained at 60 °C and 100 °C, respectively. Nitrogen was used as the carrier gas at a flow rate of 40 mL/min. CH_4_ and H_2_ concentrations in the gas samples were quantified using their respective standard concentrations (Jingao, China), with linear calibration curves ranging from 50.0 to 306 mL/L for CH_4_ and 1.01 to 19.8 mL/L for H_2_. Subsequently, the concentrations of gaseous methane and hydrogen were converted into dissolved methane (dCH_4_) and dissolved hydrogen (dH_2_) using the following equations:

1 $$\:\begin{array}{cccc}&\:{C}_{dgas}=({\alpha\:}_{gas}+{V}_{g}/{V}_{l})&\:&\:\text{}\text{}\end{array}$$

2 $$\begin{aligned}{\alpha\:}_{C{H}_{4}}&=\mathrm{e}\mathrm{x}\mathrm{p}[28.7314\times\:\mathrm{l}\mathrm{n}(T/100)\\&+101.4953\times\:(100/T)-68.8862]\end{aligned}$$

3 $$\begin{aligned}{\alpha\:}_{{H}_{2}}&=\mathrm{e}\mathrm{x}\mathrm{p}[20.1709\times\:\mathrm{l}\mathrm{n}(T/100)\\&+65.0368\times\:(100/T)-47.8948]\end{aligned}$$

where *C*_*dgas*_ refers to the concentration of the target gas (dH_2_, µM; or dCH_4_, mM) dissolved in the rumen fluid samples; *V*_*g*_ represents gas volume (L) at equilibrium after the extraction of dissolved gases, assuming the gas phase in the 20-mL syringe is at 1 atm; *V*_*l*_ refers to liquid volume (L); *T* denotes temperature in K (273 + temperature in °C) [24]; and *α*_*gas*_ refers to Bunsen absorption coefficient (L/L) for CH_4_ or H_2_ [25].

#### Estimation of Gibbs free energy of glucose conversion and methanogenesis

To assess the thermodynamic feasibility of metabolic hydrogen flows, Gibbs free energy (∆G) for six representative reactions involved in glucose conversion and methanogenesis were estimated as follows:

Glucose conversion pathways (Reaction 1 - Reaction 5):

4 $$\begin{aligned}{\mathrm C}_6{\mathrm H}_{12}{\mathrm O}_6\;&\rightarrow\;0.66\;{\mathrm C}_2{\mathrm H}_3{\mathrm O}_2\;^-\;+\;1.33\;{\mathrm C}_3{\mathrm H}_5{\mathrm O}_2\;^-\;\\&+\;0.66\;{\mathrm{CO}}_2\;+\;0.66\;{\mathrm H}_2\mathrm O\;+\;2\;\mathrm H^+\;\end{aligned}$$

5 $${\mathrm C}_6{\mathrm H}_{12}{\mathrm O}_6\;\rightarrow\;{\mathrm C}_2{\mathrm H}_3{\mathrm O}_2\;^-\;+\;{\mathrm C}_3{\mathrm H}_5{\mathrm O}_2\;^-\;+\;{\mathrm{CO}}_2\;+\;2\;\mathrm H^+\;+\;{\mathrm H}_2$$

6 $${\mathrm C}_6{\mathrm H}_{12}{\mathrm O}_6\;\rightarrow\;{\mathrm C}_4{\mathrm H}_7{\mathrm O}_2\;^-\;+\;2\;{\mathrm{CO}}_2\;+\;\mathrm H^+\;+\;2\;{\mathrm H}_2$$

7 $$\begin{aligned}{\mathrm C}_6{\mathrm H}_{12}{\mathrm O}_6\;&+0.66\;{\mathrm H}_2\mathrm O\rightarrow0.66\;{\mathrm C}_2{\mathrm H}_3{\mathrm O}_2\;^-\;\\&+\;0.66\;{\mathrm C}_4{\mathrm H}_7{\mathrm O}_2\;^-\;+\;2{\mathrm{CO}}_2\;\\&+\;1.33\mathrm H^+\;+2.66{\mathrm H}_2\;\end{aligned}$$

8 $${\mathrm C}_6{\mathrm H}_{12}{\mathrm O}_6\;+\;2\;{\mathrm H}_2\mathrm O\;\rightarrow\;2\;{\mathrm C}_2{\mathrm H}_3{\mathrm O}_2\;^-+\;2\;{\mathrm{CO}}_2\;+\;2\;\mathrm H^+\;+\;4\;{\mathrm H}_2$$

Methanogenesis (Reaction 6):

9 $${\mathrm{CO}}_2\;+\;4\;{\mathrm H}_2\;\rightarrow\;{\mathrm{CH}}_4\;+\;2\;{\mathrm H}_2\mathrm O$$

The Gibbs free energy for each reaction was computed as:

10 $$\:\begin{array}{cccc}&\:{\Delta\:}G={\Delta\:}G^\circ\:+RT\mathrm{l}\mathrm{n}\left(\frac{[Products{]}_{p}}{[Reactants{]}_{r}}\right)\end{array}$$

where *ΔG°* = standard Gibbs free energy at 298 K; *R* = 8.314 J mol⁻¹ K⁻¹; *T* = temperature (K); square brackets denote activities of solutes (molar concentration) or gases (partial pressure, atm), each raised to its stoichiometric coefficient. Reactions with ΔG < 0 are thermodynamically feasible; ΔG = 0 indicates equilibrium; ΔG > 0 denotes non-spontaneity [16, 26].

#### Rumen microbial DNA extraction and amplicon sequencing

Total microbial DNA was extracted from the rumen fluid using the microbial DNA Isolation Kit (Foregene, Chengdu, China) according to the manufacturer’s instructions. DNA quality was verified by 1% agarose gel electrophoresis. PCR amplification was performed using the following primer sets: bacteria, Ba9f (GAGTTTGATCMTGGCTCAG) and Ba515Rmod1 (CCGCGGCKGCTGGCAC) targeting 16 S rRNA; archaea, r915aF (AGGAATTGGCGGGGGAGCAC) and Ar1386R (GCGGTGTGTGCAAGGAGC) targeting 16 S rRNA; anaerobic fungi, MN100F (TCCTACCCTTTGTGAATTTG) and MNGM2 (CTGCGTTCTTCATCGTTGCG) targeting internal transcribed spacer (ITS); and protozoa, Reg841F (GACTAGGGATTGGAGTGG) and Reg1302R (AATTGCAAAGATCTATCCC) targeting 18 S rRNA [12, 27].

PCR products were visualized on 2% agarose gels, purified using the AxyPrep DNA Gel Extraction Kit (Axygen, San Francisco, USA), and quantified using the QuantiFluor™-ST Blue Fluorescence System (Promega, Beijing, China). Sequencing libraries were prepared using the TruSeq DNA Sample Preparation Kit (Bioo Scientific, Austin, TX, USA) by Majorbio Bio-Pharm Technology Co., Ltd. (Shanghai, China). The amplicon libraries were sequenced on an Illumina MiSeq platform using a paired-end 2 × 300 bp sequencing strategy at the same facility.

#### Taxonomy assignment and diversity analyses

Sequencing data were processed using the QIIME 2 pipeline [28]. After quality control and chimera removal, amplicon sequence variants (ASV) were generated using DADA 2 [29]. Taxonomic classification was performed using: SILVA 138 database for bacteria [30], RIM-DB for archaea [31], and UNITE database for fungi [32, 33]. Few protozoa taxa were found through the ASV analysis, as this method fits better for bacterial, archaeal, and fungal determination [32, 33]. Protozoal taxonomy was assigned using the Protozoa database [27] after clustering representative OTUs at 97% similarity. Clean reads were rarefied to the lowest sequencing depth within each dataset (6,520 bacterial, 7,033 archaeal, 27,786 fungal, and 22,205 protozoan sequences) to minimize biases caused by uneven sequencing depth. The rarefied data were then used to calculate α-diversity indices and β-diversity.

The community diversity was calculated using Shannon and Simpson indices, while community richness was assessed using the Chao1, ACE, and Sobs indices. The Sobs index represents the observed number of OTUs/ASVs detected in each sample [34]. In addition, β-diversity was determined using principal coordinates analysis (PCoA) based on Bray-Curtis distance using the vegan package in R Studio [35]. In each age group, grouping lines were drawn based on the geom_encircle function of the ggalt package. Taxa exhibiting a relative abundance of less than 0.01% across all samples were categorized as ‘Others’.

Linear Discriminant Analysis Effect Size (LEfSe) (http://huttenhower.sph.harvard.edu/lefse) was applied to detect differentially abundant taxa between the 0.5-year-old and 2-year-old goat groups. A significance level of *P* < 0.05 and an LDA score threshold of 2.0 were used [36].

#### Microbial metabolic pathway analysis

Rumen microbial functional potentials were predicted using PICRUSt 2 online Galaxy version [37]. Normalized ASV tables were used to infer metagenome, which were then categorized into Encyclopedia of Genes and Genomes (KEGG) pathways and visualized with the STAMP software.

### Statistical analysis

All data are presented as mean ± standard error. Differences in fermentation characteristics and Gibbs energy changes between age groups were evaluated using the independent-samples *t*-test. Microbial community differences were assessed using the Wilcoxon rank-sum test. Significance was defined as *P* < 0.05; 0.05 ≤ *P* < 0.10 was considered a trend.

Correlations among fermentation parameters, microbial abundances, and KEGG pathway levels (Level 2) were calculated [38] and plotted using Hmisc [39] and ggplot2 [40] packages in R Studio (version 1.3.1073, R Foundation for Statistical Computing, Vienna, Austria).

## Results

### Rumen fermentation profiles

Rumen fermentation parameters differed markedly between the two age groups (Table 1). The concentrations of dissolved methane, dissolved hydrogen, and total SCFAs were significantly higher in the 2-year-old goats than in the 0.5-year-old goats (*P* = 0.002). Among individual SCFAs, acetate, propionate, butyrate, isobutyrate, and isovalerate were all increased significantly with age (*P* < 0.05). However, rumen pH and acetate-to-propionate (A: P) ratio did not differ significantly between groups (*P* > 0.05) (Table 1).

**Table 1:** Comparison of rumen fermentation characteristics between 0.5-year-old and 2-year-old groups

Items	Age group		*P*-value
	0.5y	2y	
Dissolved CH_4_ (mmol/L)	0.080 ± 0.012	0.112 ± 0.005	0.035
Dissolved H_2_ (µmol/L)	0.910 ± 0.215	2.32 ± 0.447	0.018
Total SCFAs (mM)^1^	47.9 ± 2.89	62.9 ± 2.28	0.002
Acetate (mM)	30.4 ± 1.87	37.3 ± 1.49	0.017
Propionate (mM)	10.2 ± 0.68	13.5 ± 0.47	0.003
Butyrate (mM)	5.01 ± 0.43	8.30 ± 0.505	< 0.001
Valerate (mM)	0.530 ± 0.012	0.965 ± 0.050	< 0.001
Isobutyrate (mM)	0.677 ± 0.011	1.19 ± 0.029	< 0.001
Isovalerate (mM)	1.03 ± 0.01	1.75 ± 0.03	< 0.001
Acetate : Propionate	2.98 ± 0.04	2.77 ± 0.12	0.138
pH	6.84 ± 0.037	6.86 ± 0.058	0.757

0.5y: 0.5-year-old group (*n* = 6); 2y: 2-year-old group (*n* = 6)

^1^ SCFAs: short-chain fatty acids

### Microbial community composition

**Fig. 1:**
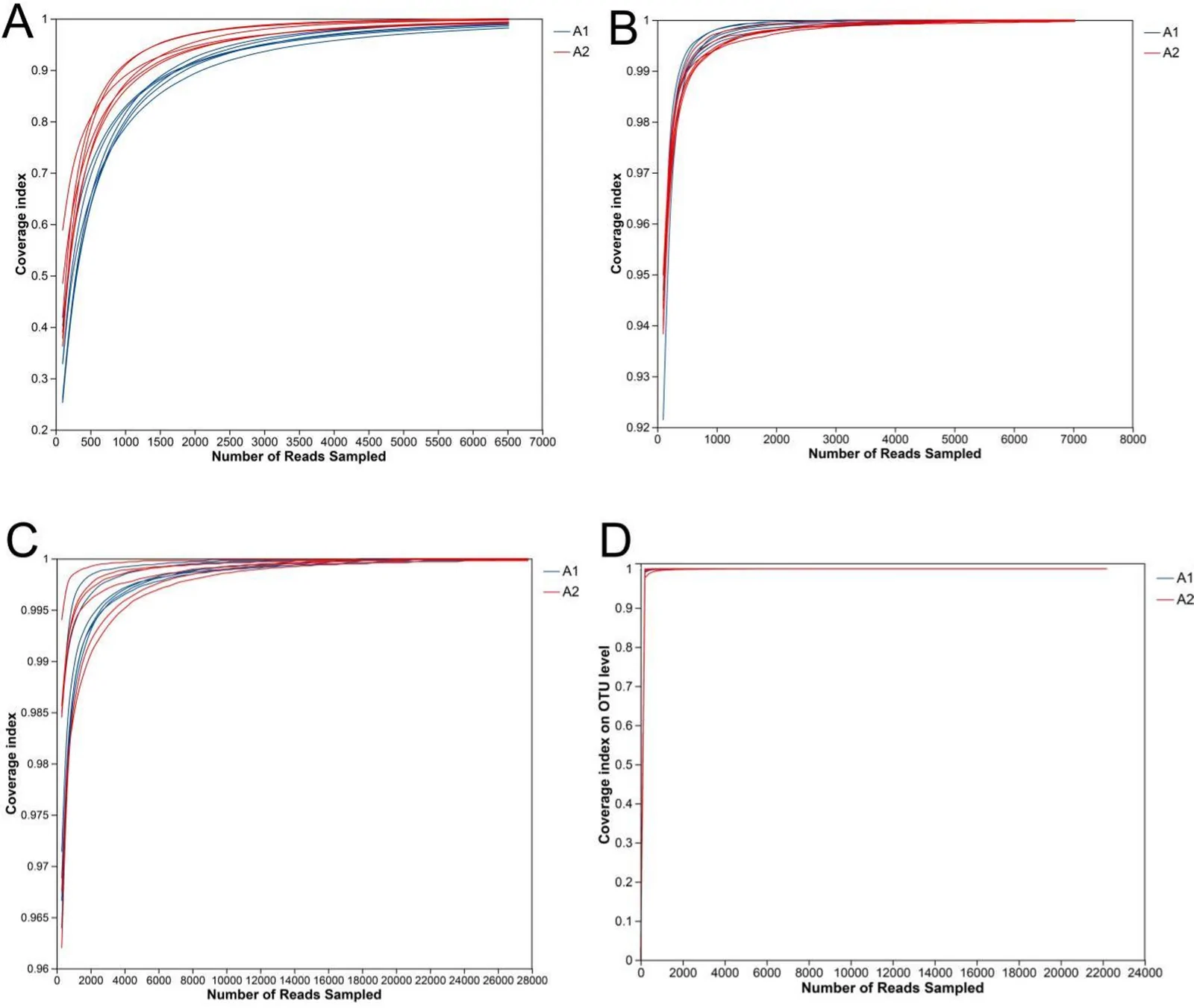
Coverage curves of the rumen microbial community. **A** Bacteria; (**B**) Archaea; (**C**) Fungi; (**D**) Protozoa. A1, 0.5-year-old group; A2, 2-year-old group

**Fig. 2:**
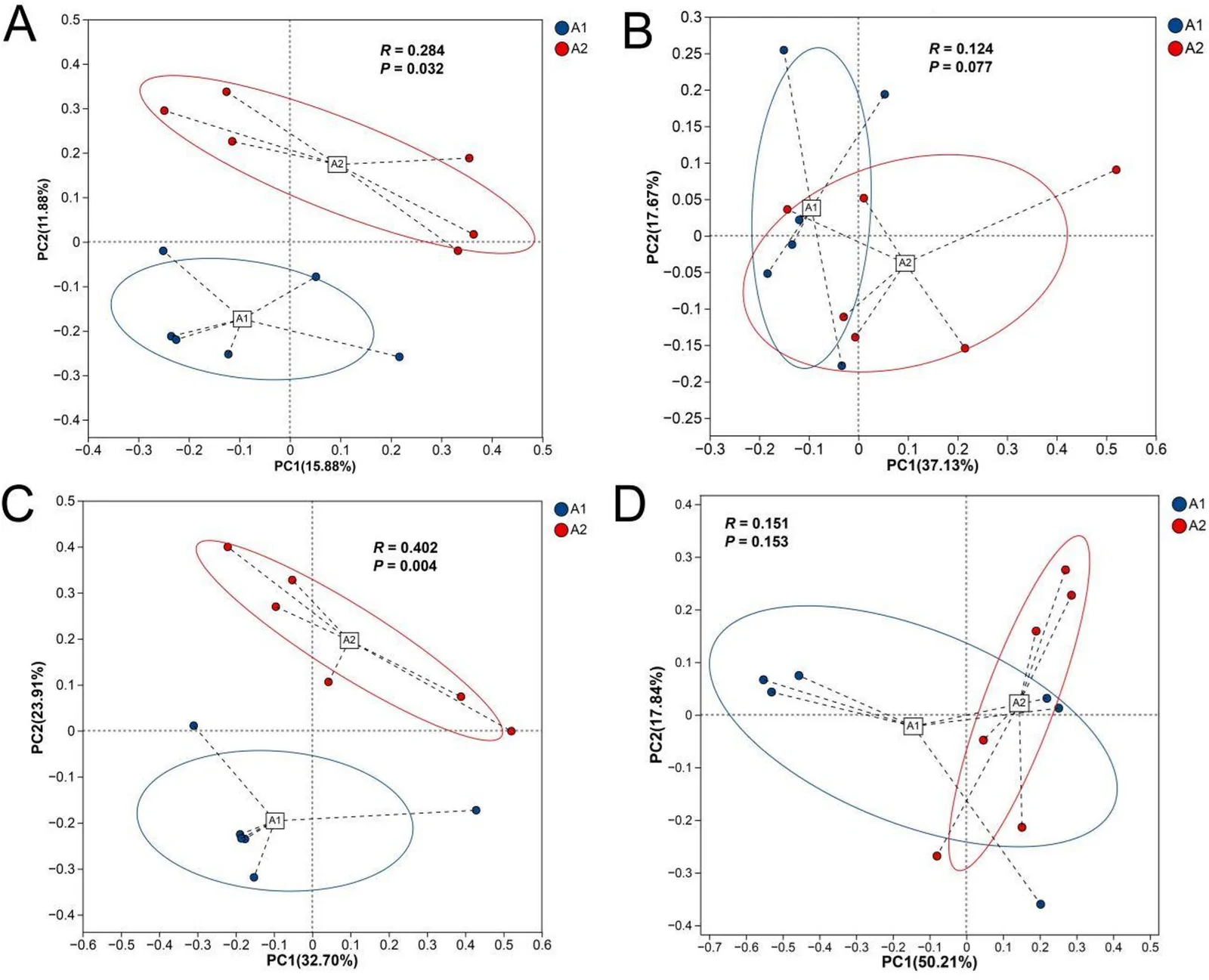
Principal coordinate analysis (PCoA) based on the Bray–Curtis distance matrix. **A** Bacteria; (**B**) Archaea; (**C**) Fungi; (**D**) Protozoa. A1, 0.5-year-old group; A2, 2-year-old group

Rarefaction and coverage curves for bacteria, archaea, fungi, and protozoa plateaued in both age groups, indicating sufficient sequencing depth (Fig. 1). Bacterial α-diversity indices (Shannon, ACE, Chao 1, and Sobs) were significantly higher in the 0.5-year-old group compared to the 2-year-old group (*P* < 0.01; Table 2). In contrast, the Simpson index tended to be greater in the older goats (*P* = 0.059). For archaea, the ACE, Chao 1, and Sobs indices were significantly higher in the 2-year-old group compared to the 0.5-year-old group (*P* < 0.05). For protozoa, the Simpson index showed a higher trend in the 0.5-year-old group (*P* = 0.068). However, no significant differences in fungal and protozoal α-diversity were observed between the two age groups (Table 2). Principal Coordinate Analysis (PCoA) based on Bray–Curtis showed clear clustering of bacterial (*R* = 0.284; *P* = 0.032) and fungal communities (*R* = 0.402; *P* = 0.004) according to age group (Fig. 2). In contrast, archaeal and protozoal communities did not exhibit distinct clustering between the two groups.

**Table 2:** Comparison of α-diversity indices of the rumen bacterial community between the 0.5-year-old and 2-year-old groups

Diversity	Bacteria		*P*-value	Archaea		*P*-value	Fungi		*P*-value	Protozoa		*P*-value
	0.5y	2y		0.5y	2y		0.5y	2y		0.5y	2y	
Shannon	5.88 ± 0.081	5.19 ± 0.165	0.003	2.14 ± 0.07	2.49 ± 0.06	0.308	2.46 ± 0.066	2.55 ± 0.150	0.567	1.06 ± 0.261	1.48 ± 0.109	0.173
Simpson	0.005 ± 0.0008	0.02 ± 0.006	0.059	0.194 ± 0.021	0.130 ± 0.010	0.482	0.130 ± 0.009	0.124 ± 0.024	0.820	0.543 ± 0.109	0.310 ± 0.035	0.068
Ace	723 ± 28.4	447 ± 26.8	< 0.001	30.4 ± 1.73	37.2 ± 1.88	0.023	61.9 ± 8.37	57.9 ± 11.9	0.785	17.7 ± 3.74	14.1 ± 2.66	0.453
Chao 1	722 ± 27.9	446 ± 26.3	< 0.001	30.2 ± 1.70	37.0 ± 1.87	0.039	61.8 ± 8.30	57.7 ± 11.9	0.784	13.4 ± 1.97	12.8 ± 2.68	0.871
Sobs	684 ± 21.6	436 ± 24.4	< 0.001	30.2 ± 1.70	36.8 ± 1.80	0.021	61.7 ± 8.27	57.2 ± 11.8	0.761	12.2 ± 1.58	12.7 ± 2.73	0.877

0.5y: 0.5-year-old group (*n* = 6); 2y: 2-year-old group (*n* = 6)

### Taxonomic composition

**Fig. 3:**
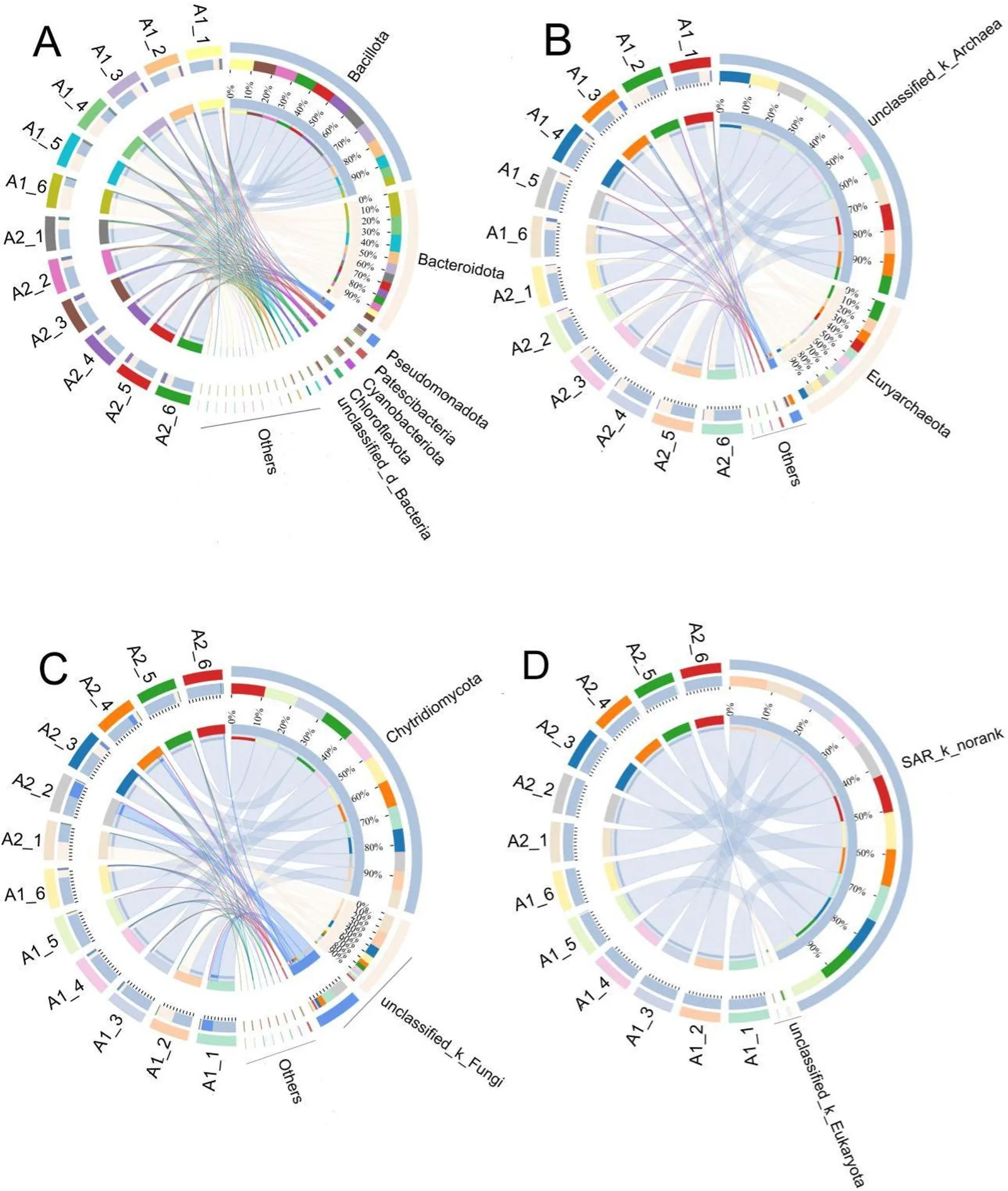
Composition and relative abundance of different phyla in the ruminal microbial community. **A** Bacteria; (**B**) Archaea; (**C**) Fungi; (**D**) Protozoa. A1, 0.5-year-old group; A2, 2-year-old group

**Fig. 4:**
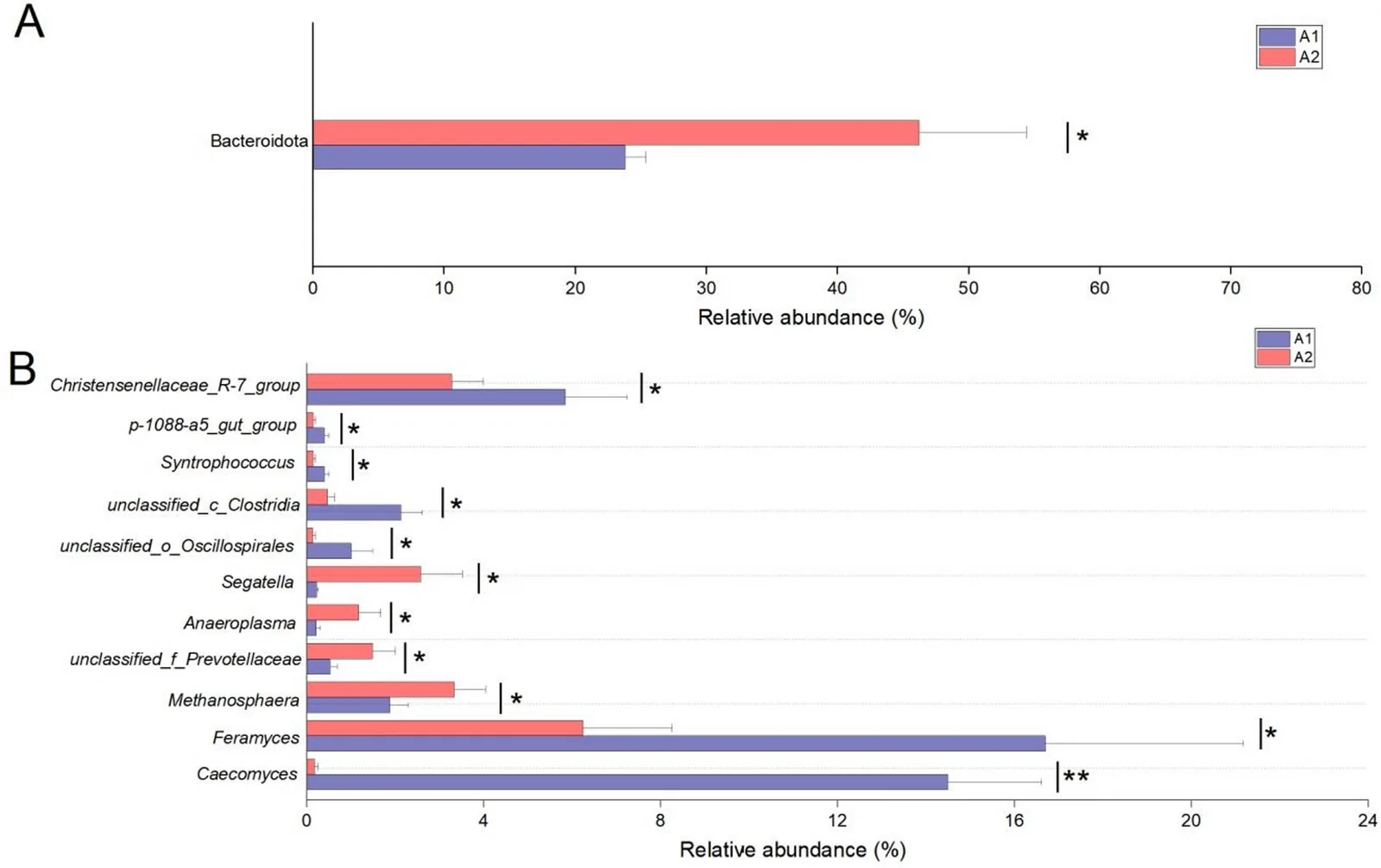
Bar plots showing the relative abundances of significantly differential microbial communities at the phylum and genus levels. **A** Bacterial phylum level; (**B**) Microbial genus level. A1, 0.5-year-old group; A2, 2-year-old group. Statistically significant differences are indicated by asterisks (^*^P < 0.05, ^**^P < 0.01, ^***^P < 0.001)

At the phylum level, bacterial communities were dominated by Bacillota, Bacteroidota, Pseudomonadota, Patescibacteria, Cyanobacteriota, Chloroflexota, and unclassified_k_norank_d_Bacteria across both groups (Fig. 3A). The archaeal community consisted mainly of phyla Euryarchaeota and unclassified_k_norank_d_Archaea (Fig. 3B). The dominant fungal phyla included Chytridiomycota, and unclassified_k_Fungi, while protozoal communities were composed mainly of SAR_k_norank, unclassified_d_Eukaryota, and unclassified_k_Animalia (Fig. 3C and D). Among bacterial phyla, Bacteroidota was significantly more abundant in the 2-year-old group compared to the 0.5-year-old group (46.2% vs. 23.8%; *P* = 0.045) (Fig. 4A). Conversely, Bacillota (65.5% vs. 47.4%; *P* = 0.065), unclassified_k_norank_d_Bacteria (1.12% vs. 0.23%; *P* = 0.054), and other phyla (1.56% vs. 1.00%; *P* = 0.093) tended to be more abundant in the 0.5-year-old group (Supplementary Table 2).

**Fig. 5:**
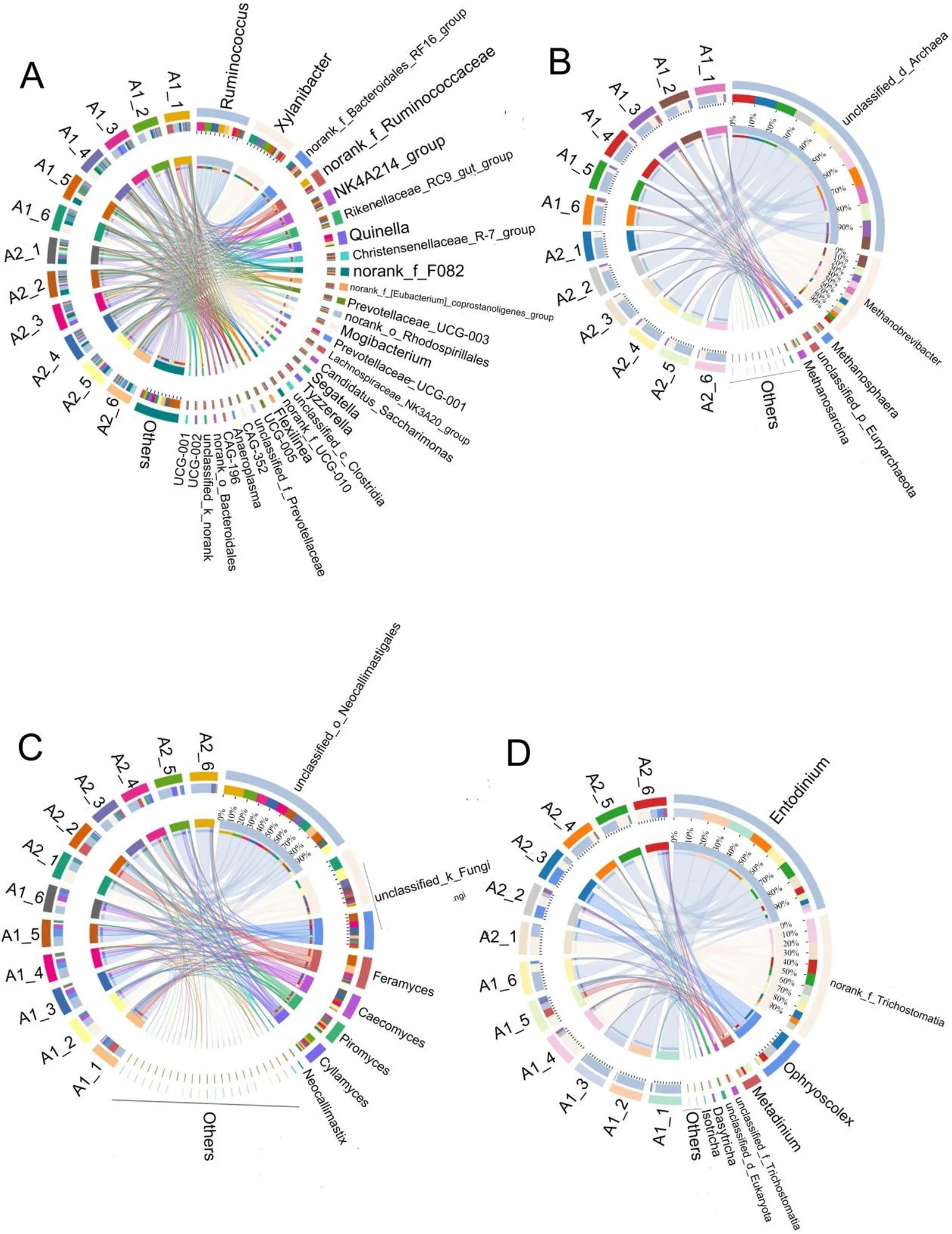
Composition and relative abundances of different genera in the ruminal microbial community. **A** Bacteria; (**B**) Archaea; (**C**) Fungi; (**D**) Protozoa. A1, 0.5-year-old group; A2, 2-year-old group

At the genus level, bacterial communities were dominated by *Ruminococcus*, *Xylanibacter*, *norank_f_Bacteroidales_RF16_group*, *norank_f_Ruminococcaceae*, *NK4A214_group*, *Rikenellaceae_RC9_gut_group*, *Quinella*, *Christensenellaceae_R-7_group*, *norank_f_F082*, *norank_f_[Eubacterium]_coprostanoligenes_group*, and *Prevotellaceae_UCG-003* (Fig. 5A). Genera *Christensenellaceae_R-7_group* (3.65% vs. 1.51%), *p-1088-a5_gut_group* (0.403% vs. 0.144%), *Syntrophococcus* (0.401% vs. 0.146%), *unclassified_c_Clostridia* (2.13% vs. 0.475%) and *unclassified_o_Oscillospirales* (1.01% vs. 0.133%) were significantly more abundant in the 0.5-year-old group (*P* < 0.05). In contrast, Genera *Segatella* (2.58% vs. 0.23%), *Anaeroplasma* (1.18% vs. 0.22%), and *unclassified_f_Prevotellaceae* (1.50% vs. 0.52%) were 11.2-fold, 5.4-fold and 2.88-fold higher, respectively, in the 2-year-old group compared to the 0.5-year-old group (*P* < 0.05) (Fig. 4B), whereas *NK4A214_group* (5.85% vs. 3.28%), *unclassified_f_Selenomonadaceae* (0.73% vs. 0.40%), *Family_XIII_AD3011_group* (0.58% vs. 0.20%), *unclassified_k_norank_d_Bacteria* (1.12% vs. 0.23%), and *Tyzzerella* (2.21% vs. 0.60%) tended to be higher in the younger goats (0.05 < *P* < 0.1) (Supplementary Table 3).

The archaeal community was dominated by *Methanobrevibacter*, *Methanosphaera*, *unclassified_k_norank_d_Archaea*, *unclassified_p_Euryarchaeota*, *Methanosarcina*, and *Others*. Compared with the 0.5-year-old group, *Methanosphaera* was more abundant in the 2-year-old group (*P* = 0.043) (Fig. 4B). *Methanosarcina* and *Others* were also significantly more abundant in the 2-year-old group (*P* < 0.05) (Supplementary Table 3).

No significant differences were observed at the phylum level in fungal and protozoa communities between the two age groups (Supplementary Table 2). The dominated fungal genera included *unclassified_o_Neocallimastigales*, *unclassified_k_Fungi*, *Feramyces*, *Caecomyces*, *Piromyces*, *Cyllamyces*, *Phascolarctobacterium*, *Neocallimastix*, and *Others* (Fig. 5C and D). Genera *Feramyces* (16.7% vs. 6.25%) and *Caecomyces* (14.5% vs. 0.179%) were significantly abundant in the 0.5-year-old group (*P* < 0.05) (Fig. 4B), while *Cyllamyces* showed a tendency of abundant in the 2-year-old group (8.81% vs. 3.48%; *P* = 0.093). Dominant protozoal genera included *Entodinium*, *norank_f_Trichostomatia*, *Metadinium*, *Ophryoscolex*, *unclassified_d_Eukaryota*, *Isotricha*, and *Others* (Fig. 5D), but none differed significantly between age groups (Supplementary Table 3).

### Biomarker taxa

**Fig. 6:**
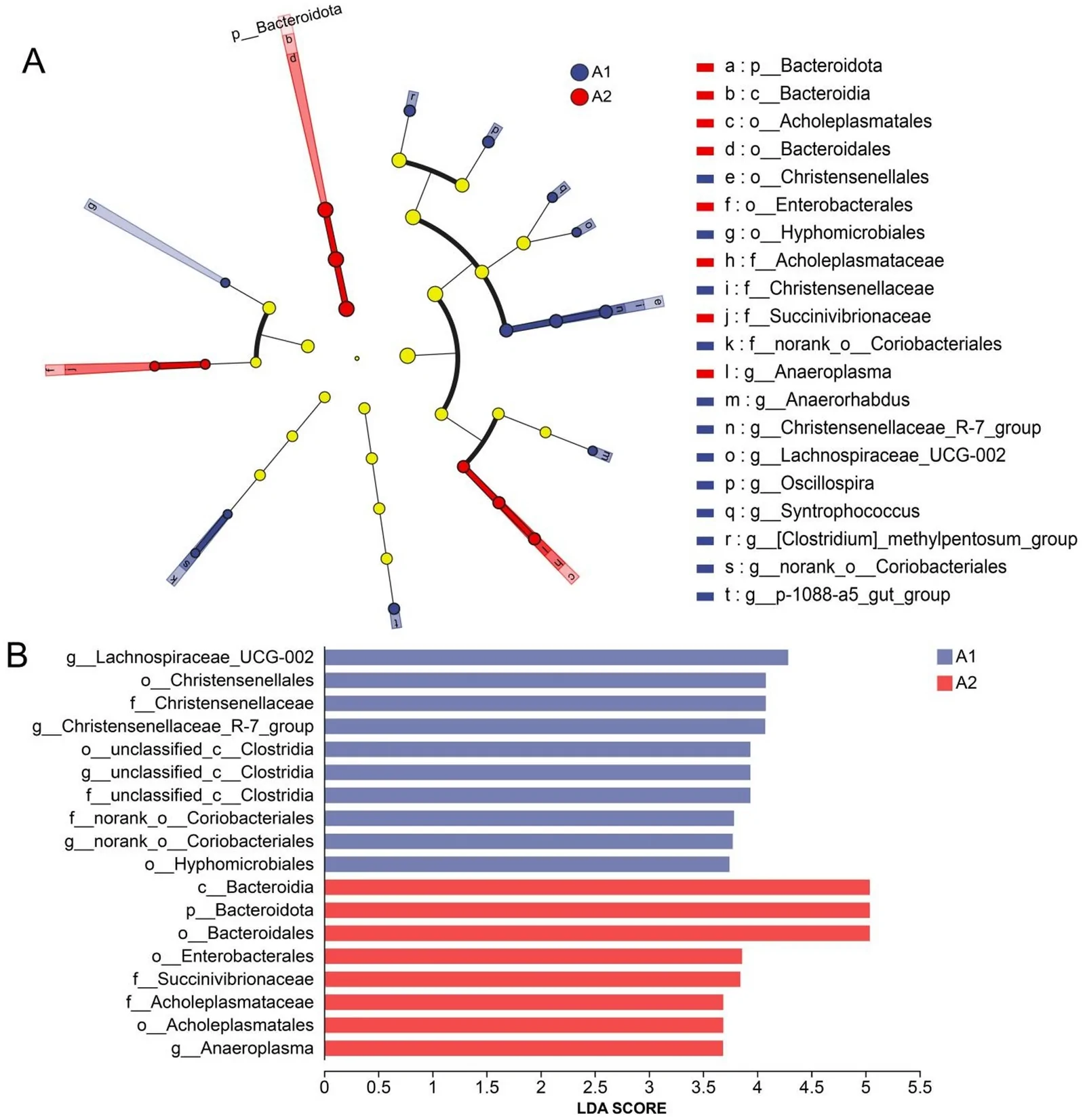
**A** Bacterial cladogram illustrating the differential taxa in Cohort 1. Taxa enriched in the 0.5-year-old group are shown in blue, and those enriched in the 2-year-old group are shown in red. The brightness of each dot corresponds to its effect size. **B** Linear discriminant analysis (LDA) coupled with effect size (LEfSe) identifies significantly enriched taxa shown in panel (A) (Continued in Fig. 7). A1, 0.5-year-old group; A2, 2-year-old group

**Fig. 7:**
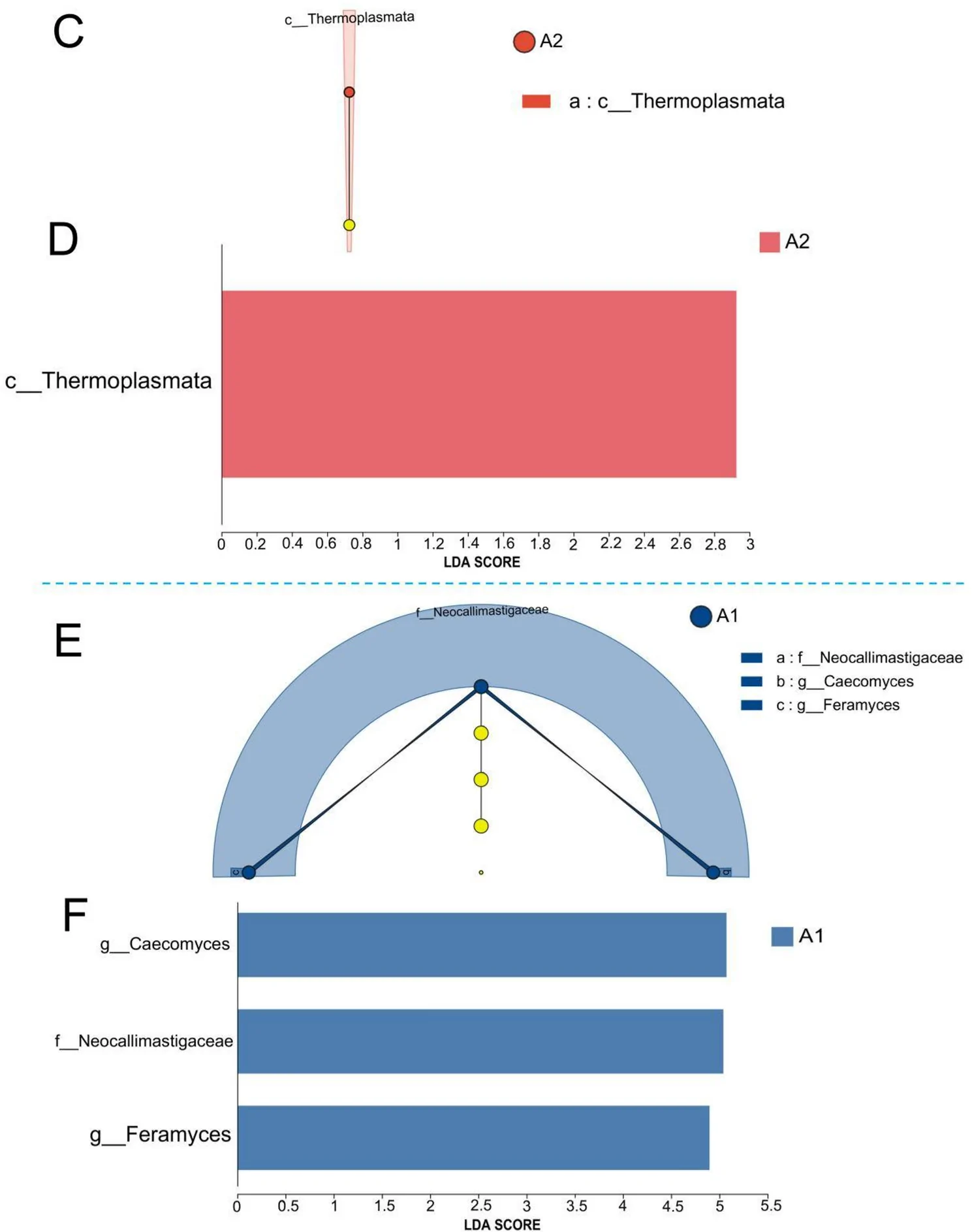
Archaeal (**C**) and fungal (**E**) cladogram representation of data from Cohort 1. Linear discriminant analysis (LDA) coupled with effect size (LEfSe) identifies significantly enriched archaeal (**D**) and fungal (**F**) taxa. Taxa enriched in the 0.5-year-old group are shown in blue, and those enriched in the 2-year-old group are shown in red. A1, 0.5-year-old group; A2, 2-year-old group (red)

Hierarchical and LDA (Linear Discriminant Analysis) analysis were used to identify bacterial, archaeal, fungal, and protozoal biomarkers (Figs. 6 and 7). In the 0.5-year-old goats, bacterial biomarkers were *Lachnospiraceae_UCG-002*, Christensenellales, Christensenellaceae, *Christensenellaceae_R-7_group*, unclassified_c_Clostridia, *unclassified_c_Clostridia*, unclassified_c_Clostridia, norank_o_Coriobacteriales, *norank_o_Coriobacteriales*, and Hyphomicrobiales, while in the 2-year-old goats, they were Bacteroidia, Bacteroidota, Bacteroidales, Enterobacterales, Succinivibrionaceae, Acholeplasmataceae, Acholeplasmatales, and *Anaeroplasma*. For archaea, only *Thermoplasmata* was identified as a biomarker in the 2-year-old goats. Among fungi, *Caecomyces*, Neocallimastigaceae, and *Feramyces* were enriched in younger goats. No protozoal biomarkers were detected.

### Estimation of Gibbs energy changes and microbial metabolic pathways

All glucose conversion pathways (Reactions 1–5) and the methanogenesis pathway (Reaction 6) exhibited negative ΔG values (ΔG < 0), indicating thermodynamic feasibility under ruminal conditions. The ΔG values for Reactions 2–5 were significantly higher (less exergonic) in the 2-year-old goats (*P* < 0.05), whereas methanogenesis (Reaction 6) showed a significantly lower ΔG (*P* = 0.01), suggesting a stronger thermodynamic drive for methane formation in older goats. Reaction 1 showed no significant difference between age groups (Table 3).

**Table 3:** Comparison of Estimated Gibbs energy changes of various rumen pathways between 0.5-year-old and 2-year-old groups

Reaction	ΔG (kJ/mol reaction)		*P*-value
	0.5y	2y	
Reaction 1: C_6_H_12_O_6_ → 0.66 C_2_H_3_O_2_^-^ + 1.33 C_3_H_5_O_2_^།^ + 0.66 CO_2_ + 0.66 H_2_O + 2 H^+^	-266 ± 0.423	-264 ± 0.764	0.058
Reaction 2: C_6_H_12_O_6_ → C_2_H_3_O_2_^-^ + C_3_H_5_O_2_^།^ + CO_2_ + 2 H^+^ + H_2_	-265 ± 0.338	-261 ± 0.620	< 0.001
Reaction 3: C_6_H_12_O_6_ → C_4_H_7_O_2_^-^ + 2 CO_2_ + H^+^ + 2 H_2_	-285 ± 1.08	-279 ± 1.14	0.006
Reaction 4: C_6_H_12_O_6_ +0.66 H_2_O→0.66 C_2_H_3_O_2_^-^ + 0.66 C_4_H_7_O_2_^།^ + 2 CO_2_ + 1.33 H^+^ +2.66 H_2_	-228 ± 1.56	-220 ± 1.43	0.004
Reaction 5: C_6_H_12_O_6_ + 2 H_2_O → 2 C_2_H_3_O_2_^-^+ 2 CO_2_ + 4 H_2_+ 2 H^+^	-187 ± 2.24	-177 ± 2.71	0.024
Reaction 6: CO_2_ + 4 H_2_ → CH_4_ + 2 H_2_O	-67.7 ± 2.22	-76.5 ± 1.95	0.014

0.5y: 0.5-year-old group (*n* = 6); 2y: 2-year-old group (*n* = 6)

**Fig. 8:**
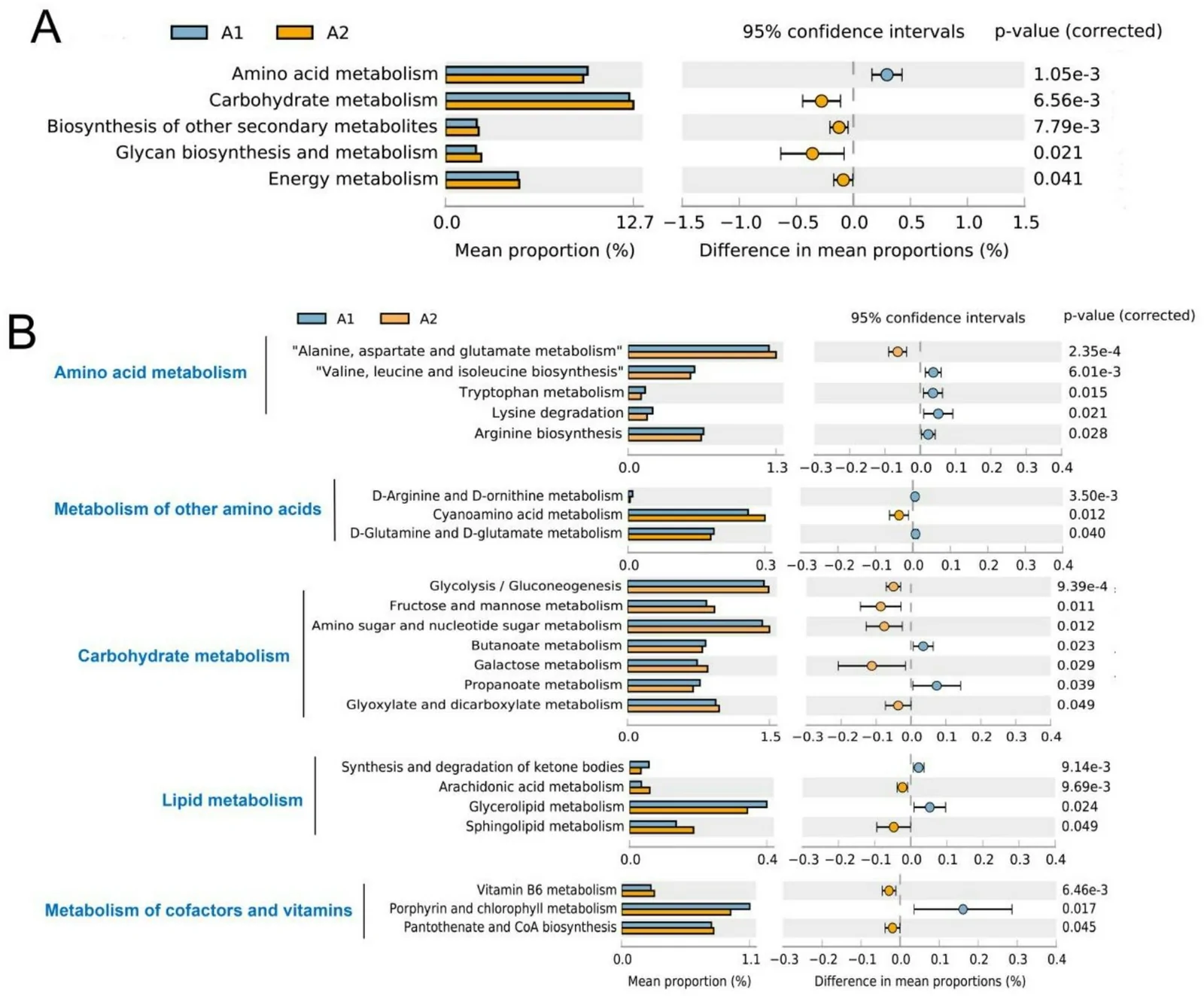
Extended bar plots showing predicted rumen microbial functions based on PICRUSt 2 at the second (**A**) and third (**B**) levels of the KEGG pathway. A1, 0.5-year-old group; A2, 2-year-old group

PICRUSt 2 functional predictions revealed significant age-related differences in microbial metabolic capacities (Fig. 8). In younger goats, amino acid metabolism pathways were enriched (*P* < 0.05; Fig. 8A), whereas in older goats, pathways associated with carbohydrate metabolism, glycan biosynthesis and metabolism, and energy metabolism were enriched (*P* < 0.05).

At KEGG Level 3, the 0.5-year-old group showed enrichment in pathways for valine, leucine, and isoleucine biosynthesis, tryptophan metabolism, lysine degradation, and arginine biosynthesis, whereas the 2-year-old group was enriched in alanine, aspartate, and glutamate metabolism (Fig. 8B). Within the category metabolism of other amino acids, the D-arginine and D-ornithine metabolism and D-glutamine and D-glutamate metabolism pathways were enriched in the 0.5-year-old group, whereas the cyanoamino acid metabolism pathway was reduced compared to the 2-year-old group.

In carbohydrate metabolism, glycolysis/gluconeogenesis, fructose and mannose metabolism, amino sugar and nucleotide sugar metabolism, galactose metabolism, and glyoxylate and dicarboxylate metabolism showed higher predicted relative abundance in the 2-year-old goats (*P* < 0.05). In contrast, butanoate metabolism and propanoate metabolism pathways were enriched in the 0.5-year-old goats. In lipid metabolism, synthesis and degradation of ketone bodies and glycerolipid metabolism were more abundant in younger goats, whereas arachidonic acid metabolism and sphingolipid metabolism pathways were relatively more represented in older goats. Within the category of cofactor and vitamin metabolism, the 2-year-old group showed higher predicted abundances of pathways related to vitamin B6 metabolism and pantothenate and CoA biosynthesis, while porphyrin and chlorophyll metabolism was relatively enriched in the 0.5-year-old group.

### Correlation between fermentation parameters, microbes, and metabolic pathways

**Fig. 9:**
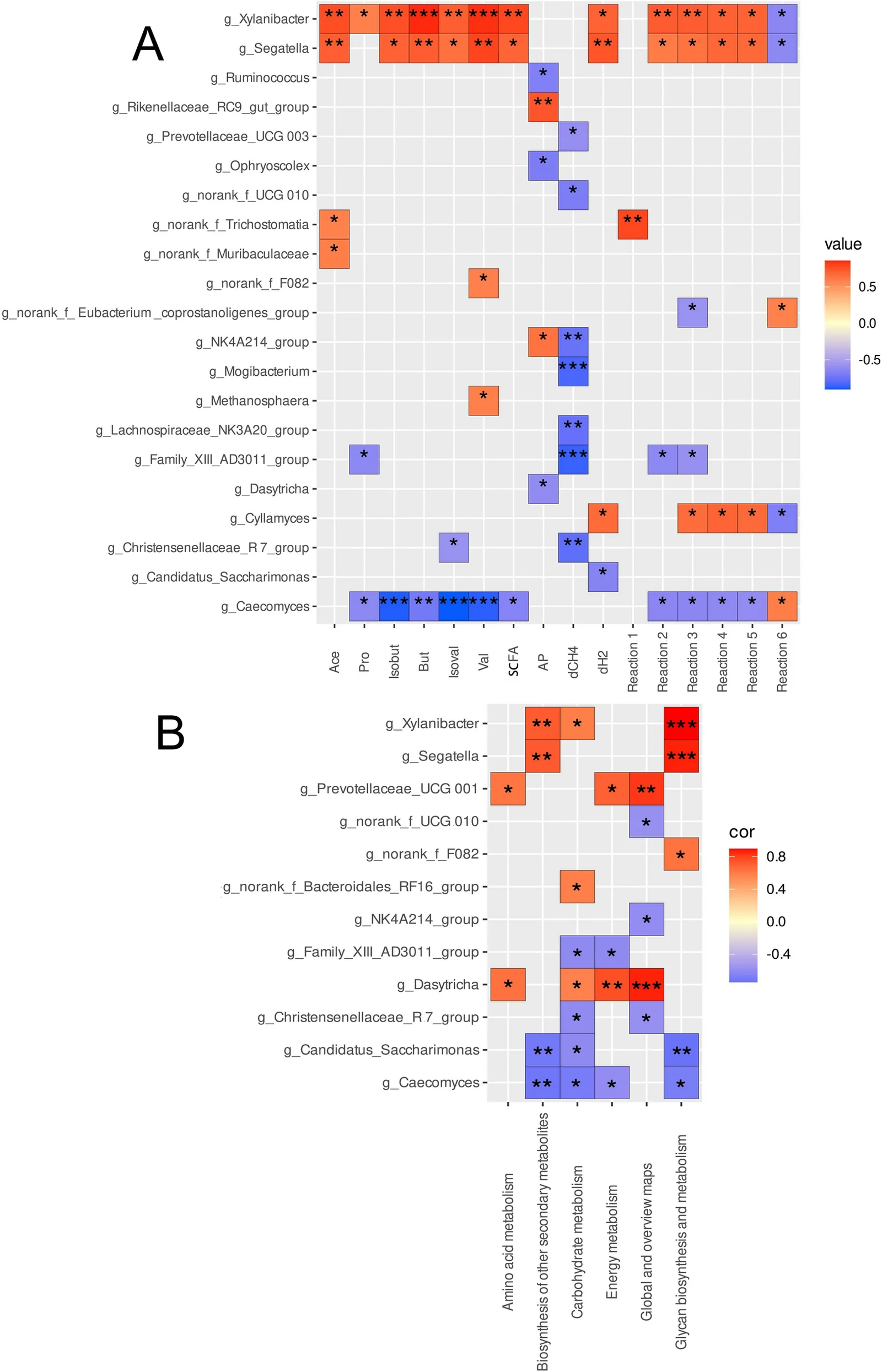
Spearman’s correlation of rumen bacteria with rumen fermentation parameters (**A**) and microbial KEGG level 2 functional modules (**B**). Only significant correlations are shown (* *P* < 0.05, **P < 0.01, ***P < 0.001)

Correlation analyses revealed significant correlations between specific microbial genera, fermentation end products, and metabolic pathways (Fig. 9). *Xylanibacter* was positively correlated with acetate, propionate, butyrate, isobutyrate, valerate, isovalerate, total SCFA, dH_2_ and ΔG for Reactions 2 − 5 (glucose conversion pathways), but negatively correlated with Reaction 6 (methanogenesis) (*P* < 0.05; Fig. 9A). *Segatella* showed similar positive correlations with acetate, isobutyrate, butyrate, isovalerate, valerate, total SCFA, dH_2_ and ΔG for Reactions 2 − 5 (*P* < 0.05). *Cyllamyces* displayed positive correlations with dH_2_ and ΔG for Reactions 3 − 5, but a negative correlation with Reaction 6 (*P* < 0.05) (Fig. 9A).

*Ruminococcus*, *Ophryoscolex*, and *Dasytricha* were negatively correlated with the acetate-to-propionate (A: P) ratio, whereas *Rikenellaceae_RC9_gut_group* and *NK4A214_group* showed a positive correlation. *Norank_f_F082* and *Methanosphaera* were positively correlated with valerate, while *norank_f_Trichostomatia* and *norank_f_Muribaculaceae* were positively correlated with acetate. *Norank_f_Trichostomatia* also showed a positive correlation with Reaction 1. *Eubacterium_coprostanoligenes_group* correlated positively with Reaction 6, whereas *Family_XIII_AD3011_group* was negatively correlated with Reaction 2. *Christensenellaceae_R-7_group*, *Candidatus_Saccharimonas*, and *Family_XIII_AD3011_group* were negatively correlated with isovalarate, dH_2_, and propionate, respectively. Several genera, including *Prevotellaceae_UCG-003*, *norank_f_UCG-010*, *NK4A214_group*, *Mogibacterium*, *Lachnospiraceae_NK3A20_group*, *Family_XIII_AD3011_group*, and *Christensenellaceae_R-7_group* displayed negative correlations with dCH_4_. *Caecomyces* showed negative correlations with propionate, butyrate, isobutyrate, valerate, isovalerate, total SCFA, and Reactions 2–5, but a positive correlation with Reaction 6 (Fig. 9A).

*Prevotellaceae_UCG-001* and *Dasytricha* were positively correlated with amino acid metabolism pathways (Fig. 9B), whereas *Xylanibacter* and *Segatella* were positively correlated, and *Candidatus_Saccharimonas* and *Caecomyces* were negatively correlated, with the biosynthesis of other secondary metabolites. *Xylanibacter*, *norank_f_Bacteroidales_RF16_group*, and *Dasytricha* were positively correlated, while *Caecomyces*, *Family_XIII_AD3011_group*, *Christensenellaceae_R-7_group*, and *Candidatus_Saccharimonas* were negatively correlated with carbohydrate metabolism. *Prevotellaceae_UCG-001* and *Dasytricha* showed positive correlations, whereas *Family_XIII_AD3011_group* and *Caecomyces* showed negative correlations with energy metabolism.

Finally, *Prevotellaceae_UCG-001* and *Dasytricha* were positively correlated, while *norank_f_UCG-010*, *NK4A214_group*, and *Christensenellaceae_R-7_group* were negatively correlated with global and overview metabolic maps. *Xylanibacter*, *norank_f_F082*, and *Segatella* were positively correlated, whereas *Candidatus_Saccharimonas* and *Caecomyces* were negatively correlated with glycan biosynthesis and metabolism (*P* < 0.05).

## Discussion

### Age-dependent shifts in rumen fermentation, hydrogen metabolism, and nutrient allocation

Rumen maturation profoundly influences hydrogen metabolism and microbial fermentation pathways. In this study, 2-year-old goats exhibited significantly higher concentrations of dissolved hydrogen (dH₂), dissolved methane (dCH₄), and short-chain fatty acids (SCFAs) than 0.5-year-old goats, indicating a more developed and active rumen fermentation system. The mature rumen likely supports enhanced carbohydrate degradation, generating more metabolic hydrogen that promotes methanogenic activity and methane production. This interpretation is supported by the enrichment of carbohydrate and energy metabolism pathways in older goats (Fig. 8A). Increased glycolytic activity and enhanced conversion of pyruvate to acetate and butyrate likely provide more reducing equivalents (H₂ or [2 H]), facilitating methanogenesis. Consistent with previous studies [41, 42], the positive association between dissolved hydrogen and SCFA concentrations suggests that hydrogen production is closely coupled with fermentation intensity. Although increased SCFA production has previously been linked to reduced ruminal pH during aging [12], this relationship was not observed in the present study, possibly due to dietary buffering, saliva secretion, and dynamic regulation of rumen acid-base homeostasis [43]. Therefore, ruminal pH alone may not accurately reflect fermentation activity, which is regulated by multiple factors, including age, diet, and physiological status.

The higher SCFA concentrations observed in adult goats likely resulted from increased feed intake and the maturation of the rumen microbial community, which together may provide greater substrate availability and enhanced fermentative capacity. However, elevated SCFA levels should not be interpreted as direct evidence of improved feed efficiency, as nutrient digestibility and metabolic flux were not measured in the present study. Despite higher dry matter intake (DMI), adult goats showed lower average daily gain (ADG) than young goats (Supplementary Table 1), reflecting a developmental shift in nutrient allocation. Young goats were in an active growth phase, during which absorbed nutrients are preferentially directed toward tissue deposition and protein accretion [44, 45]. This interpretation was supported by the enrichment of nitrogen metabolism-related microbial taxa, including *Christensenellaceae R-7 group* and *Family XIII AD3011 group*, together with enhanced amino acid metabolism pathways predicted by PICRUSt 2. In contrast, adult goats showed enrichment of Bacteroidota and carbohydrate-degrading microorganisms, such as *Segatella*, *Anaeroplasma* and *Cyllamyces*, accompanied by increased predicted carbohydrate and energy metabolism pathways. Collectively, these findings may suggest a mature rumen microbial ecosystem optimized for fiber degradation and SCFA production.

### Age-related succession of the rumen microbiome promotes functional specialization and hydrogen metabolism

Rumen microbial maturation involves substantial ecological succession, with a transition from a diverse but unstable community in young animals to a more specialized and functionally stable ecosystem in adults [12, 13]. In this study, bacterial alpha diversity indices (Shannon, Chao 1, and ACE) were higher in 0.5-year-old goats than in 2-year-old goats, suggesting an active microbial colonization and transition phase during early rumen development. However, reduced diversity in adult goats does not necessarily indicate impaired microbial function; instead, it likely reflects the selective enrichment of microorganisms better adapted to the established diet and host environment. As the rumen matures, specialized microbial groups involved in fiber degradation, carbohydrate metabolism, and hydrogen turnover become dominant, enhancing metabolic stability and fermentation efficiency [13]. Consistent with this interpretation, adult goats showed higher SCFA concentrations, indicating increased fermentative activity despite lower bacterial diversity. At the phylum level, Bacteroidota was significantly enriched in adult goats (46.2% vs. 23.8%), consistent with its important role in degrading complex polysaccharides and promoting SCFA production [46]. In contrast, Bacillota tended to be more abundant in young goats (65.5% vs. 47.4%), reflecting the transitional microbial community during early rumen establishment [12]. Genera associated with carbohydrate fermentation and fiber utilization, including *Anaeroplasma* and *unclassified Prevotellaceae* [47, 48], increased in adults, whereas nitrogen- and amino acid-associated taxa, including *Christensenellaceae R-7 group*, *Syntrophococcus*, and *Family XIII AD3011 group* [49, 50], were more abundant in young goats, indicating age-dependent differences in substrate utilization strategies.

The maturation-associated microbial shift was further reflected in fungal and archaeal community composition and their potential roles in rumen hydrogen metabolism. The tendency towards enrichment of *Cyllamyces*, a lignocellulose-degrading fungus [51], in adult goats, together with the higher abundance of *Caecomyces* in juveniles, may suggest a developmental transition from less specialized fermentation toward enhanced fiber degradation [52]. Anaerobic fungi contribute to plant cell wall breakdown and release H₂, CO₂, acetate, and formate during fermentation, and their hydrogen production may support methanogenesis through interspecies hydrogen transfer [6]. Although fungal H₂ production was not directly measured in this study, age-related shifts in fungal composition may contribute to differences in rumen hydrogen availability. Similarly, increased abundances of *Methanosarcina* and *Methanosphaera* in adult goats indicate enhanced methanogenic potential, as these archaea occupy distinct hydrogen-related metabolic niches [53, 54]. Methanogens utilize fermentation-derived hydrogen to maintain redox balance and promote methane formation, which may explain the higher dH₂ and dCH₄ concentrations observed in mature goats. Overall, the age-related restructuring of bacterial, fungal, and archaeal communities suggests that rumen development involves a shift from a growth-associated microbial ecosystem toward a specialized community optimized for fiber degradation, SCFA production, and hydrogen turnover.

### Rumen maturation alters microbial thermodynamics and hydrogen partitioning

Thermodynamics determines the energetic feasibility and direction of rumen fermentation, whereas microbial pathways govern fermentation rates and product yields. Fermentative microbes generate metabolic hydrogen during carbohydrate degradation, and the reoxidation of reduced cofactors through electron transfer is essential for sustaining fermentation [2]. The Gibbs free energy change (ΔG) provides a thermodynamic indicator of reaction feasibility and helps explain microbial responses to hydrogen availability [5, 16]. In this study, all glucose fermentation pathways (Reactions 1–5) and methanogenesis (Reaction 6) showed negative (ΔG < 0) in both age groups, indicating that these reactions were energetically favorable under rumen conditions. However, 2-year-old goats exhibited a significantly lower ΔG for methanogenesis and less favorable energy release from SCFA-producing pathways, suggesting higher dissolved H₂ in adult goats may drive H₂-producing fermentation reactions less thermodynamically favourable, while making H₂-consuming methanogenesis more favourable. The greater thermodynamic favorability of methanogenesis in adult goats was consistent with their higher hydrogen availability and increased methanogen abundance [14]. Although gas methane emissions were not directly measured, these results indicate a stronger potential for methane production in mature goats. Therefore, strategies aimed at reducing methanogenesis or redirecting metabolic hydrogen toward alternative electron sinks may be particularly relevant for adult animals. Future studies combining thermodynamic modeling with direct methane measurements are needed to validate these predictions.

KEGG functional predictions further supported the metabolic transition associated with rumen maturation. Adult goats showed enrichment of predicted carbohydrate and energy metabolism pathways, whereas young goats exhibited higher representation of amino acid metabolism pathways. The increased abundance of glycolysis/gluconeogenesis and other carbohydrate-related pathways in adults suggests enhanced carbon flow through central metabolism, providing more reducing equivalents for hydrogen production and methanogenesis. In contrast, enrichment of amino acid biosynthesis pathways, including valine, leucine, isoleucine, and tryptophan metabolism, in young goats reflects their greater requirement for microbial protein synthesis during growth. Age-related differences were also evident in predicted hydrogen-related metabolic pathways. Adult goats showed increased representation of glycolysis/gluconeogenesis, fructose and mannose metabolism, amino sugar and nucleotide sugar metabolism, galactose metabolism, and glyoxylate and dicarboxylate metabolism, suggesting greater potential for the generation of fermentation intermediates and reducing power for hydrogen generation. Conversely, young goats exhibited greater predicted enrichment of butanoate and propanoate metabolism, pathways that incorporate reducing equivalents into SCFA production and may limit hydrogen availability for methanogenesis [2]. Together, these findings demonstrate that rumen maturation may involve a shift from growth-oriented anabolic metabolism in young goats toward energy-yielding catabolic metabolism in adults, accompanied by enhanced hydrogen turnover, greater methanogenic potential, and improved predicted microbial pathways for carbohydrate and energy metabolism in the rumen ecosystem.

### Limitations and future directions

This study provides insights into age-related changes in rumen fermentation, microbial communities, and hydrogen metabolism; however, several limitations should be considered. The Gibbs free energy (ΔG) calculations were based on dissolved gas concentrations and assumed equilibrium and homogeneous rumen conditions. In reality, the rumen is a spatially and temporally dynamic ecosystem, where hydrogen concentrations vary among microhabitats and fluctuate over time [3, 55]. Therefore, the calculated ΔG values should be interpreted as theoretical estimates of thermodynamic potential rather than direct measurements of in vivo reaction conditions. Moreover, a negative ΔG indicates that a reaction is thermodynamically favorable but does not determine its actual rate, which is regulated by kinetic factors, enzyme activity, gene expression, and metabolic regulation. Future studies combining thermodynamic modeling with approaches such as RNA stable isotope probing (RNA-SIP) and metabolic inhibitor experiments could help identify active microorganisms and verify the specific pathways responsible for hydrogen production and utilization [56]. In addition, methanogenic archaea regulate hydrogen availability by maintaining a low H₂ partial pressure, thereby influencing fermentative bacteria, protozoa, and fungi through interspecies hydrogen transfer. This interaction affects the balance among acetate, propionate, butyrate, and methane production, highlighting the need for integrated studies of microbial networks rather than individual pathways alone [57].

Rumen fluid samples in this study were collected using a stomach tube, which may introduce potential bias into microbial community analysis. Compared with sampling from rumen-cannulated animals, stomach tube sampling can be influenced by sampling location, saliva contamination, selective collection of liquid-associated microorganisms, and spatial heterogeneity of microbial communities within the rumen. Previous studies have shown that sampling methods can influence the observed composition and diversity of rumen microbiota, particularly for low-abundance or compartment-specific taxa [9, 58]. Therefore, the microbial profiles reported in this study should be interpreted with consideration of these methodological limitations. Nevertheless, stomach tube sampling remains a widely used, minimally invasive approach for rumen microbiome studies in live animals. Future studies using rumen cannulation, multiple sampling sites, or complementary molecular approaches will help validate these findings and provide a more comprehensive understanding of rumen microbial ecology.

The relatively small sample size (*n* = 6 per group) represents another limitation of this study and may restrict the generalizability of these findings to broader goat populations. Therefore, the observed age-associated differences in rumen microbiota composition, fermentation characteristics, and methanogenic potential should be further validated using larger cohorts and animals raised under diverse dietary and production conditions. In addition, the Reg841F/Reg1302R primer pair used for protozoal profiling may have limited taxonomic resolution, as closely related species within certain genera cannot be reliably distinguished, potentially leading to an underestimation of protozoal diversity. Furthermore, although rarefaction was applied to normalize sequencing depth among samples, the protozoan datasets were rarefied to 22,205 sequences per sample, and only 61 OTUs were identified in this study. This relatively low number of OTUs may have reduced the detection of rare taxa; therefore, the identified OTUs should be considered a conservative representation of rumen protozoal diversity. Future investigations incorporating protozoa-specific primers, long-read sequencing technologies, and alternative normalization approaches may provide a more comprehensive characterization of rumen protozoal communities.

Overall, rumen fermentation is governed by complex interactions among microbial composition, gene expression, enzymatic activity, and metabolic pathways. Integrating metagenomics, metatranscriptomics, metabolomics, and thermodynamic analyses will be essential for elucidating the relationships between specific microorganisms and fermentation processes, ultimately facilitating the development of targeted strategies for manipulating rumen metabolism.

## Conclusions

This study provides evidence that rumen microbial community structure, metabolic hydrogen ([2 H]) redistribution, and fermentation-related thermodynamic profiles are associated with age-related changes in goats. As the rumen matures from 0.5 to 2 years, microbial community composition shifts from enrichment of amino acid-associated taxa toward a community containing taxa involved in carbohydrate and energy metabolism. This microbial transition is accompanied by changes in dissolved hydrogen and methane concentrations, more negative Gibbs free energy values for methanogenic reaction, and increased potential for interspecies hydrogen disposal.

The increased abundance of *Segatella*, *Anaeroplasma*, *unclassified Prevotellaceae*, and *Cyllamyces* in mature goats suggests their potential involvement in the age-associated development of rumen fermentation characteristics. Conversely, the higher abundance of *Christensenellaceae R-7 group*, *Syntrophococcus*, and *Caecomyces* in young goats may reflect differences in microbial ecological niches and substrate availability during early rumen development. Together, these findings suggest that rumen maturation is accompanied by coordinated changes in microbial composition, hydrogen metabolism, and fermentation energetics, with increased methane production as a potential consequence of enhanced hydrogen disposal. These observations improve our understanding of microbial and thermodynamic changes during rumen development and may contribute to future strategies aimed at modulating hydrogen metabolism to improve feed energy utilization and reduce methane emissions in ruminant production systems.

## Supplementary Information

Supplementary Material 1.

## Data Availability

All sequencing data generated in this study have been deposited in the NCBI Sequence Read Archive (SRA) database under the BioProject ID: PRJNA1333636. Additional datasets supporting the findings of this study are available from the corresponding authors upon reasonable request.

## References

[1] Jami E, Mizrahi I. Host-rumen microbiome interactions and influences on feed conversion efficiency (FCE), methane production and other productivity traits. In: Mackie RI, McSweeney C, editors. Improving Rumen Function. United Kingdom: Burleigh Dodds Science Publishing Limited; 2020. pp. 547–66. 10.19103/AS.2020.0067.18.

[2] Ungerfeld EM. Metabolic hydrogen flows in rumen fermentation: principles and possibilities of interventions. Front Microbiol. 2020;11:589. 10.3389/fmicb.2020.00589. 32351469 PMC7174568

[3] Wang K, Xiong B, Zhao X. Could propionate formation be used to reduce enteric methane emission in ruminants? Sci Total Environ. 2023;855:158867. 10.1016/j.scitotenv.2022.158867. 36122712

[4] Belanche A, Bannink A, Dijkstra J, Durmic Z, Garcia F, Santos FG, et al. Feed additives for methane mitigation: a guideline to uncover the mode of action of antimethanogenic feed additives for ruminants. J Dairy Sci. 2025;108(1):375–94. 10.3168/jds.2024-25046. 39725503

[5] Wang WW, Guo W, Jiao J, Ungerfeld EM, Jing X, Huang X, et al. Effects of ratios of yak to cattle inocula on methane production and fiber digestion in rumen in vitro cultures. J Integr Agric. 2025;24(4):1270–84. 10.1016/j.jia.2024.01.026.

[6] Mizrahi I, Wallace RJ, Moraïs S. The rumen microbiome: Balancing food security and environmental impacts. Nat Rev Microbiol. 2021;19:553–66. 10.1038/s41579-021-00543-6. 33981031

[7] Li QS, Wang R, Ma ZY, Zhang XM, Jiao JZ, Zhang ZG, et al. Dietary selection of metabolically distinct microorganisms drives hydrogen metabolism in ruminants. ISME J. 2022;16(11):2535–46. 10.1038/s41396-022-01294-9. 35931768 PMC9562222

[8] Nugrahaeningtyas E, Lee JS, Park KH. Greenhouse gas emissions from livestock: Sources, estimation, and mitigation. J Anim Sci Technol. 2024;66(6):1083–98. 10.5187/jast.2024.e86. 39691613 PMC11647415

[9] Henderson G, Cox F, Ganesh S, Jonker A, Young W, Abecia L, et al. Rumen microbial community composition varies with diet and host, but a core microbiome is found across a wide geographical range. Sci Rep. 2015;5:14567. 10.1038/srep14567. 26449758 PMC4598811

[10] Liu K, Zhang Y, Yu Z, Xu Q, Zheng N, Zhao S, et al. Ruminal microbiota-host interaction and its effect on nutrient metabolism. Anim Nutr. 2021;7:49–55. 10.1016/j.aninu.2020.12.001. 33997331 PMC8110878

[11] Fernando SC, Adams S, Lakamp A, Spangler ML. Stochastic and deterministic factors that shape the rumen microbiome. J Dairy Sci. 2025. 10.3168/jds.2024-25797. 40043768

[12] Guo W, Zhou M, Ma T, Bi S, Wang W, Zhang Y, et al. Survey of rumen microbiota of domestic grazing yak during different growth stages revealed novel maturation patterns of four key microbial groups and their dynamic interactions. Anim Microbiome. 2020;2:1–20. 10.1186/s42523-020-00042-8. PMC7807461 33499950

[13] Ahmad AA, Zhang J, Liang Z, Du M, Yang Y, Zheng J, et al. Age-dependent variations in rumen bacterial community of Mongolian cattle from weaning to adulthood. BMC Microbiol. 2022;22(1):213. 10.1186/s12866-022-02627-6. 36071396 PMC9450343

[14] Janssen PH, Kirs M. Structure of the archaeal community of the rumen. Appl Environ Microbiol. 2008;74(12):3619–25. 10.1128/AEM.02812-07. 18424540 PMC2446570

[15] Guo YQ, Liu JX, Lu Y, Zhu WY, Denman SE, et al. Effect of tea saponin on methanogenesis, microbial community structure and expression of mcrA gene in cultures of rumen microorganisms. Lett Appl Microbiol. 2008;47(5):421–6. 10.1111/j.1472-765X.2008.02459.x. 19146532

[16] Ungerfeld EM. Limits to dihydrogen incorporation into electron sinks alternative to methanogenesis in ruminal fermentation. Front Microbiol. 2015;6:1272. 10.3389/fmicb.2015.01272. 26635743 PMC4649033

[17] Ministry of Agriculture and Rural Affairs of the People’s Republic of China. Nutrient Requirements of Meat-Type Sheep and Goat: NY/T 816–2021. Beijing: China Agriculture; 2021. (In Chinese).

[18] Wang M, Wang R, Janssen PH, Zhang XM, Sun XZ, Pacheco D, et al. Sampling procedure for the measurement of dissolved hydrogen and volatile fatty acids in the rumen of dairy cows. J Anim Sci. 2016;94(3):1159–69. 10.2527/jas.2015-9658. 27065277

[19] AOAC. Official Methods of Analysis. 20th ed. West Lafayette, IN, USA: Association of Official Analytical Chemists; 2016.

[20] Van Soest PJ, Robertson JB, Lewis BA. Methods for dietary fiber, neutral detergent fiber, and nonstarch polysaccharides in relation to animal nutrition. J Dairy Sci. 1991;74:3583–97. 10.3168/jds.S0022-0302(91)78551-2. 1660498

[21] Erwin ES, Marco GJ, Emery EM. Volatile fatty acid analyses of blood and rumen fluid by gas chromatography. J Dairy Sci. 1961;44:1768–71. 10.3168/jds.S0022-0302(61)89956-6.

[22] Wang WW, Ungerfeld EM, Degen AA, Jing X, Guo W, Zhou J, et al. Ratios of rumen inoculum from Tibetan and Small-tailed Han sheep influenced in vitro fermentation and digestibility. Anim Feed Sci Technol. 2020;267:114562. 10.1016/j.anifeedsci.2020.114562.

[23] Zhang ZG, Xu D, Wang L, Hao J, Wang J, Zhou X, et al. Convergent evolution of rumen microbiomes in high-altitude mammals. Curr Biol. 2016;26(14):1873–9. 10.1016/j.cub.2016.05.012. 27321997

[24] Wang M, Sun XZ, Janssen PH, Tang SX, Tan ZL. Responses of methane production and fermentation pathways to the increased dissolved hydrogen concentration generated by eight substrates in in vitro ruminal cultures. Anim Feed Sci Technol. 2014;194:1–11. 10.1016/j.anifeedsci.2014.04.012.

[25] Wiesenburg DA, Guinasso NL Jr. Equilibrium solubilities of methane, carbon monoxide, and hydrogen in water and sea water. J Chem Eng Data. 1979;24(4):356–60. 10.1021/je60083a006.

[26] Wang M, Ungerfeld EM, Wang R, Zhou CS, Basang ZZ, Ao SM, Tan ZL. Supersaturation of dissolved hydrogen and methane in rumen of Tibetan sheep. Front Microbiol. 2016;7:850. 10.3389/fmicb.2016.00850. 27379028 PMC4906022

[27] Kittelmann S, Kirk MR, Jonker A, McCulloch A, Janssen PH. Buccal swabbing as a noninvasive method to determine bacterial, archaeal, and eukaryotic microbial community structures in the rumen. Appl Environ Microbiol. 2015;81(21):7470–83. 10.1128/AEM.02385-15. 26276109 PMC4592876

[28] Bolyen E, Rideout JR, Dillon MR, Bokulich NA, Abnet CC, Al-Ghalith GA, et al. Reproducible, interactive, scalable and extensible microbiome data science using QIIME 2. Nat. Biotechnol. 2019;37(8):852–7. 10.1038/s41587-019-0209-9. PMC7015180 31341288

[29] Callahan BJ, McMurdie PJ, Rosen MJ, Han AW, Johnson AJA, Holmes SP. DADA2: High-resolution sample inference from Illumina amplicon data. Nat Methods. 2016;13(7):581–3. 10.1038/nmeth.3869. 27214047 PMC4927377

[30] Quast C, Pruesse E, Yilmaz P, Gerken J, Schweer T, Yarza P, et al. The SILVA ribosomal RNA gene database project: improved data processing and web-based tools. Nucleic Acids Res. 2013;41(D1):D590–6. 10.1093/nar/gks1219. 23193283 PMC3531112

[31] Seedorf H, Kittelmann S, Henderson G, Janssen PH. RIM-DB: a taxonomic framework for community structure analysis of methanogenic archaea from the rumen and other intestinal environments. PeerJ. 2014;2:e494. 10.7717/peerj.494. 25165621 PMC4137658

[32] Fregulia P, Campos MM, Dias RJP, Liu J, Guo W, Pereira LGR, et al. Taxonomic and predicted functional signatures reveal linkages between the rumen microbiota and feed efficiency in dairy cattle raised in tropical areas. Front Microbiol. 2022;13:1025173. 10.3389/fmicb.2022.1025173. 36523842 PMC9745175

[33] Weinroth MD, Belk AD, Dean C, Noyes N, Dittoe DK, Rothrock MJ Jr, et al. Considerations and best practices in animal science 16S ribosomal RNA gene sequencing microbiome studies. J Anim Sci. 2022;100(2):skab346. 10.1093/jas/skab346. 35106579 PMC8807179

[34] Schloss PD, Gevers D, Westcott SL. Reducing the effects of PCR amplification and sequencing artifacts on 16S rRNA-based studies. PLoS ONE. 2011;6(12):e27310. 10.1371/journal.pone.0027310. 22194782 PMC3237409

[35] Oksanen J, Simpson GL, Blanchet FG, Kindt R, Legendre P, Minchin PR, et al. vegan: Community Ecology Package. R package version 2.7-1. Vienna: Comprehensive R Archive Network (CRAN); 2025. 10.32614/CRAN.package.vegan.

[36] Li F, Hitch TCA, Chen Y, Creevey CJ, Guan LL. Multi-omics reveals that the rumen microbiome and its metabolome together with the host metabolome contribute to individualized dairy cow performance. Microbiome. 2020;8:64. 10.1186/s40168-020-00819-8. 32398126 PMC7218573

[37] Douglas GM, Maffei VJ, Zaneveld JR, Yurgel SN, Brown JR, Taylor CM, et al. PICRUSt 2 for prediction of metagenome functions. Nat Biotechnol. 2020;38(6):685–8. 10.1038/s41587-020-0548-6. 32483366 PMC7365738

[38] Kyawt YY, Aung M, Xu Y, Sun Z, Zhou Y, Zhu W, et al. Dynamic changes of rumen microbiota and serum metabolome revealed increases in meat quality and growth performances of sheep fed bio-fermented rice straw. J Anim Sci Biotechnol. 2024;15(1):34. 10.1186/s40104-023-00983-5. 38419130 PMC10900626

[39] Harrell FE Jr. Hmisc: Harrell Miscellaneous. R package version 5.2-6. Vienna: Comprehensive R Archive Network (CRAN); 2026. 10.32614/CRAN.package.Hmisc.

[40] Wickham H, Chang W, Henry L, Pedersen TL, Takahashi K, Wilke C, Woo K, Yutani H, Dunnington D. ggplot2: Elegant Graphics for Data Analysis. R package version 4.0.3. 2026. Available from: https://ggplot2.tidyverse.org/.

[41] Ellis JL, Dijkstra J, Kebreab E, Bannink A, Odongo NE, McBride BW, et al. Aspects of rumen microbiology central to mechanistic modelling of methane production in cattle. J Agric Sci. 2008;146(2):213–33. 10.1017/S0021859608007752.

[42] Wang M, Wang R, Xie TY, Janssen PH, Sun XZ, Beauchemin KA, et al. Shifts in rumen fermentation and microbiota are associated with dissolved ruminal hydrogen concentrations in lactating dairy cows fed different types of carbohydrates. J Nutr. 2016;146(9):1714–21. 10.3945/jn.116.232462. 27511925

[43] Amirault K, Wright R, Sujani S, Dos Reis BR, Osorio J, Fernandes T, et al. Ruminal pH sensing for monitoring volatile fatty acid concentrations in response to short-term dietary disruption. JDS Commun. 2024;5(2):91–5. 10.3168/jdsc.2023-0409. 38482114 PMC10928433

[44] Nolan JV, et al. Nutrient requirements of domesticated ruminants. Collingwood, Australia: CSIRO Publishing; 2007.

[45] National Research Council. Nutrient Requirements of Small Ruminants: Sheep, Goats, Cervids, and New World Camelids. Washington, DC: National Academies; 2007.

[46] Jami E, Israel A, Kotser A, Mizrahi I. Exploring the bovine rumen bacterial community from birth to adulthood. ISME J. 2013;7(6):1069–79. 10.1038/ismej.2013.2. 23426008 PMC3660679

[47] Belanche A, Yáñez-Ruiz DR, Detheridge AP, Griffith GW, Kingston-Smith AH, Newbold CJ. Maternal versus artificial rearing shapes the rumen microbiome having minor long-term physiological implications. Environ Microbiol. 2019;21:4360–77. 10.1111/1462-2920.14801. 31518039 PMC6899609

[48] Huang S, Ji S, Suen G, Wang F, Li S. The rumen bacterial community in dairy cows is correlated to production traits during freshening period. Front Microbiol. 2021;12:630605. 10.3389/fmicb.2021.630605. 33746924 PMC7969525

[49] Liu C, Wu H, Liu S, Chai S, Meng Q, Zhou Z. Dynamic alterations in yak rumen bacteria community and metabolome characteristics in response to feed type. Front Microbiol. 2019;10:1116. 10.3389/fmicb.2019.01116. 31191470 PMC6538947

[50] Arce-Cordero JA, Fan P, Monteiro HF, Dai X, Jeong KC, Faciola AP. Effects of choline chloride on the ruminal microbiome at 2 dietary neutral detergent fiber concentrations in continuous culture. J Dairy Sci. 2022;105(5):4128–43. 10.3168/jds.2021-21591. 35282921

[51] Fliegerova K, Kaerger K, Kirk P, Voigt K. Rumen fungi. In: Puniya AK, Singh R, Kamra DN, editors. Rumen microbiology: From evolution to revolution. New Delhi, India: Springer; 2015. pp. 97–112. 10.1007/978-81-322-2401-3_7.

[52] Gordon GLR. Selection of anaerobic fungi for better fibre degradation in the rumen. In: Akin DE, Ljungdahl LG, Wilson JR, Harris PJ, editors. Microbial and plant opportunities to improve lignocellulose utilization by ruminants. New York: Elsevier; 1990. pp. 301–9. 10.1016/S0168-6445(03)00072-X.

[53] Thauer RK, Kaster AK, Seedorf H, Buckel W, Hedderich R. Methanogenic archaea: Ecologically relevant differences in energy conservation. Nat Rev Microbiol. 2008;6:579–91. 10.1038/nrmicro1931. 18587410

[54] Tang Y, Liu X, Zhu S, Jia M, Liu JX, Sun HZ. New insights into enteric methane production based on the archaeal genome atlas of the ruminant gastrointestinal tract. J Adv Res. 2024;74:13. 10.1016/j.jare.2024.09.016. 39426464 PMC12302423

[55] Boone DR, Johnson RL, Liu Y. Diffusion of the interspecies electron carriers H2 and formate in methanogenic ecosystems and its implications in the measurement of Km for H2 or formate uptake. Appl Environ Microbiol. 1989;55(7):1735–41. 10.1128/aem.55.7.1735-1741.1989. 16347966 PMC202943

[56] Godwin S, Kang A, Gulino LM, Manefield M, Gutierrez-Zamora ML, Kienzle M, et al. Investigation of the microbial metabolism of carbon dioxide and hydrogen in the kangaroo foregut by stable isotope probing. ISME J. 2014;8(9):1855–65. 10.1038/ismej.2014.25. 24621520 PMC4139718

[57] Abecia L, Martínez-Fernandez G, Waddams K, Martín-García AI, Pinloche E, Creevey CJ, et al. Analysis of the rumen microbiome and metabolome to study the effect of an antimethanogenic treatment applied in early life of kid goats. Front Microbiol. 2018;9:2227. 10.3389/fmicb.2018.02227. 30356690 PMC6189281

[58] de Assis Lage CF, Räisänen SE, Melgar A, Nedelkov K, Chen X, Oh J, et al. Comparison of two sampling techniques for evaluating ruminal fermentation and microbiota in the planktonic phase of rumen digesta in dairy cows. Front Microbiol. 2020;11:618032. 10.3389/fmicb.2020.618032. 33424820 PMC7785721

