# Supplementary material for: Age-related shifts of dissolved hydrogen and its impact on rumen fermentation dynamics and microbial communities in goats (Capra hircus)
Source: BMC Microbiol. 2026 Aug 27;26:778. doi: 10.1186/s12866-026-05563-x (PMC13540943; doi:10.1186/s12866-026-05563-x)
Supplement: Supplementary file 1 [file 12866_2026_5563_MOESM1_ESM.docx]

**Supplementary Table 1** Comparison of dry matter intake and growth performance between 0.5- and 2-year-old goats

| Items | Age group | | *P*-value |
| --- | --- | --- | --- |
|  | 0.5y | 2y |  |
| DMI (kg DM/day)^1^ | 0.847 ± 0.015 | 1.21 ± 0.025 | <0.001 |
| ADG (g/day)^2^ | 132 ± 3.82 | 66.7 ± 2.47 | <0.001 |
| DMI / BW^0.75^ | 0.071 ± 0.0016 | 0.071 ± 0.0015 | 0.904 |

0.5y: 0.5-year-old group (n= 6); 2y: 2-year-old group (n= 6).

^1^ DMI: average daily dry matter intake; ^2^ ADG: average daily gain.

In the calculation of BW^0.75^, BW was defined as the average body weight, calculated as the mean of the initial and final body weights.

**Supplementary Table 2** Relative abundances of rumen microbial communities at the phylum level in two age groups

| Phylum (%) | 0.5-year-old | 2-year-old | *P*-value |
| --- | --- | --- | --- |
| Bacteria | | | |
| Bacillota | 65.5 ± 1.10 | 47.4 ± 7.57 | 0.065 |
| Bacteroidota | 23.8 ± 1.55 | 46.2 ± 8.17 | 0.045 |
| Pseudomonadota | 3.19 ± 0.820 | 1.71 ± 0.370 | 0.199 |
| Patescibacteria | 1.75 ± 0.181 | 1.43 ± 0.348 | 0.575 |
| Cyanobacteriota | 1.89 ± 0.429 | 1.14 ± 0.339 | 0.240 |
| Chloroflexota | 1.23 ± 0.402 | 0.928 ± 0.332 | 0.689 |
| unclassified_k_norank_d_Bacteria | 1.12 ± 0.613 | 0.228 ± 0.143 | 0.054 |
| Others^1^ | 1.56 ± 0.221 | 0.999 ± 0.220 | 0.093 |
| Archaea | | | |
| unclassified_d_Archaea | 72.6 ± 1.98 | 70.6 ± 5.04 | 0.997 |
| Euryarchaeota | 27.2 ± 2.05 | 28.4 ± 5.04 | 0.937 |
| Others^1^ | 0.293 ± 0.141 | 1.78 ± 0.486 | 0.004 |
| Fungi |  |  |  |
| Chytridiomycota | 75.9 ± 6.16 | 69.7 ± 7.33 | 0.589 |
| unclassified_k_Fungi | 23.4 ± 6.10 | 29.9 ± 7.30 | 0.522 |
| Others^1^ | 0.722 ± 0.249 | 0.297 ± 0.113 | 0.229 |
| Protozoa | | | |
| SAR_k_norank | 99.1 ± 0.733 | 99.2 ± 0.492 | 0.872 |
| unclassified_d_Eukaryota | 0.941 ± 1.79 | 0.771 ± 0.482 | 0.872 |
| Others^1^ | 0 | 0.010 ± 0.009 | 0.405 |

^1^ Others: taxa with relative abundances less than 0.01% across all samples were classified as "Others".

**Supplementary Table 3** Relative abundances of rumen microbial communities at the genus level in two age groups

| Genus (%) | 0.5-year-old | 2-year-old | *P*-value |
| --- | --- | --- | --- |
| Bacteria | | | |
| *Ruminococcus* | 17.7 ± 2.99 | 16.7 ± 6.01 | 0.937 |
| *Xylanibacter* | 7.12 ± 0.668 | 21.4 ± 5.17 | 0.180 |
| *Quinella* | 5.30 ± 2.74 | 2.82 ± 2.12 | 0.336 |
| *norank_f_F082* | 2.29 ± 0.241 | 2.80 ± 0.333 | 0.485 |
| *Rikenellaceae_RC9_gut_group* | 4.24 ± 1.08 | 4.23 ± 1.72 | 0.575 |
| *Christensenellaceae_R-7_group* | 3.65 ± 0.781 | 1.51 ± 0.273 | 0.026 |
| *p-1088-a5_gut_group* | 0.403 ± 0.082 | 0.144 ± 0.045 | 0.043 |
| *Syntrophococcus* | 0.401 ± 0.077 | 0.146 ± 0.025 | 0.041 |
| *unclassified_c_Clostridia* | 2.13 ± 0.475 | 0.475 ± 0.156 | 0.012 |
| *unclassified_o_Oscillospirales* | 1.01 ± 0.476 | 0.133 ± 0.065 | 0.016 |
| *NK4A214_group* | 5.85 ± 1.39 | 3.28 ± 0.716 | 0.093 |
| *unclassified_f_Selenomonadaceae* | 0.726 ± 0.088 | 0.396 ± 0.123 | 0.065 |
| *Family_XIII_AD3011_group* | 0.575 ± 0.167 | 0.204 ± 0.068 | 0.064 |
| *unclassified_k_norank_d_Bacteria* | 1.12 ± 0.613 | 0.228 ± 0.143 | 0.054 |
| *Tyzzerella* | 2.21 ± 0.737 | 0.598 ± 0.284 | 0.094 |
| *norank_f_Bacteroidales_RF16_group* | 4.79 ± 1.01 | 6.18 ± 1.79 | 0.937 |
| *norank_f_Ruminococcaceae* | 4.39 ± 1.09 | 5.35 ± 4.31 | 0.128 |
| *Flexilinea* | 1.22 ± 0.404 | 0.920 ± 0.335 | 0.699 |
| *Prevotellaceae_UCG-003* | 1.99 ± 0.827 | 1.81 ± 0.257 | 0.394 |
| *norank_f_[Eubacterium]_coprostanoligenes_group* | 2.77 ± 0.682 | 1.623 ± 0.314 | 0.310 |
| *Mogibacterium* | 2.35 ± 0.775 | 1.03 ± 0.292 | 0.132 |
| *Lachnospiraceae_NK3A20_group* | 2.14 ± 0.769 | 1.17 ± 0.299 | 0.336 |
| *Segatella* | 0.228 ± 0.038 | 2.67 ± 0.738 | 0.046 |
| *Anaeroplasma* | 0.220 ± 0.097 | 1.176 ± 0.494 | 0.044 |
| *unclassified_f_Prevotellaceae* | 0.524 ± 0.143 | 1.49 ± 0.313 | 0.048 |
| *Candidatus_Saccharimonas* | 1.70 ± 0.168 | 1.12 ± 0.416 | 0.240 |
| *norank_o_Rhodospirillales* | 2.44 ± 0.717 | 1.19 ± 0.285 | 0.180 |
| *Prevotellaceae_UCG-001* | 1.19 ± 0.281 | 2.19 ± 1.53 | 0.699 |
| *norank_f_UCG-010* | 1.58 ± 0.483 | 0.757 ± 0.158 | 0.240 |
| *CAG-352* | 1.24 ± 0.437 | 0.706 ± 0.335 | 0.485 |
| *UCG-005* | 0.900 ± 0.385 | 1.132 ± 0.780 | 0.484 |
| *Acetitomaculum* | 0.483 ± 0.194 | 0.368 ± 0.128 | 0.818 |
| *Selenomonas* | 0.120 ± 0.050 | 0.212 ± 0.097 | 0.871 |
| *UCG-002* | 0.741 ± 0.162 | 0.598 ± 0.237 | 0.470 |
| *UCG-001* | 0.312 ± 0.128 | 0.984 ± 0.404 | 0.128 |
| *CAG-196* | 0.833 ± 0.151 | 0.547 ± 0.171 | 0.309 |
| *UCG-004* | 0.516 ± 0.367 | 0.320 ± 0.133 | 0.873 |
| *Anaerorhabdus* | 0.458 ± 0.065 | 0.570 ± 0.129 | 0.394 |
| Others^1^ | 11.4 ± 0.822 | 9.58 ± 2.45 | 0.240 |
| Archaea | | | |
| *unclassified_d_Archaea* | 72.8 ± 2.05 | 70.6 ± 5.04 | 0.996 |
| *Methanobrevibacter* | 23.4 ± 1.94 | 23.2 ± 4.57 | 0.699 |
| *Methanosphaera* | 1.88 ± 0.419 | 3.34 ± 0.304 | 0.043 |
| *unclassified_p_Euryarchaeota* | 1.91 ± 0.494 | 1.81 ± 0.552 | 0.999 |
| *Methanosarcina* | 0.00 | 0.135 ± 0.057 | 0.028 |
| Others^1^ | 0.009 ± 0.006 | 0.201 ± 0.117 | 0.019 |
| Fungi | | | |
| *unclassified_o_Neocallimastigales* | 35.7 ± 2.43 | 46.9 ± 7.15 | 0.240 |
| *unclassified_k_Fungi* | 23.4 ± 6.10 | 29.9 ± 7.30 | 0.589 |
| *Feramyces* | 16.7 ± 4.47 | 6.25 ± 2.01 | 0.043 |
| *Caecomyces* | 14.5 ± 2.11 | 0.179 ± 0.084 | 0.005 |
| *Piromyces* | 5.33 ± 0.639 | 7.28 ± 1.21 | 0.179 |
| *Cyllamyces* | 3.48 ± 1.34 | 8.81 ± 2.49 | 0.093 |
| *Neocallimastix* | 0.248 ± 0.139 | 0.292 ± 0.187 | 0.929 |
| Others^1^ | 0.723 ± 0.337 | 0.297 ± 0.087 | 0.531 |
| Protozoa | | | |
| *Entodinium* | 62.5 ± 14.9 | 36.9 ± 9.82 | 0.309 |
| *norank_f_Trichostomatia* | 26.4 ± 11.6 | 37.4 ± 5.76 | 0.240 |
| *Ophryoscolex* | 2.81 ± 1.22 | 19.7 ± 10.0 | 0.809 |
| *Metadinium* | 5.41 ± 4.09 | 3.08 ± 1.43 | 0.935 |
| *unclassified_f_Trichostomatia* | 1.75 ± 1.63 | 0.894 ± 0.608 | 0.744 |
| *unclassified_d_Eukaryota* | 0.941 ± 0.733 | 0.771 ± 0.482 | 0.872 |
| *Dasytricha* | 0.074 ± 0.067 | 0.949 ± 0.933 | 0.924 |
| *Isotricha* | 0.143 ± 0.104 | 0.296 ± 0.296 | 0.753 |
| Others^1^ | 0.001 ± 0.001 | 0.010 ± 0.010 | 0.934 |

^1^ Others: taxa with relative abundances less than 0.01% across all samples were classified as "Others".

Where CAG and UCG groups represent uncultured or provisional taxa.
